# Experience-dependent emergence of beta and gamma band oscillations in the primary visual cortex during the critical period

**DOI:** 10.1038/srep17847

**Published:** 2015-12-09

**Authors:** Guang Chen, Malte J. Rasch, Ran Wang, Xiao-hui Zhang

**Affiliations:** 1 Institute of Neuroscience, State Key Laboratory of Neuroscience, Shanghai Institutes for Biological Sciences, Chinese Academy of Sciences, Shanghai 200031, China; 2 State Key Laboratory of Cognitive Neuroscience & Learning, IDG/McGovern Institute for Brain Research, Beijing Normal University, Beijing 100875, China; 3 University of Chinese Academy of Sciences, Shanghai, China

## Abstract

Neural oscillatory activities have been shown to play important roles in neural information processing and the shaping of circuit connections during development. However, it remains unknown whether and how specific neural oscillations emerge during a postnatal critical period (CP), in which neuronal connections are most substantially modified by neural activity and experience. By recording local field potentials (LFPs) and single unit activity in developing primary visual cortex (V1) of head-fixed awake mice, we here demonstrate an emergence of characteristic oscillatory activities during the CP. From the pre-CP to CP, the peak frequency of spontaneous fast oscillatory activities shifts from the beta band (15–35 Hz) to the gamma band (40–70 Hz), accompanied by a decrease of cross-frequency coupling (CFC) and broadband spike-field coherence (SFC). Moreover, visual stimulation induced a large increase of beta-band activity but a reduction of gamma-band activity specifically from the CP onwards. Dark rearing of animals from the birth delayed this emergence of oscillatory activities during the CP, suggesting its dependence on early visual experience. These findings suggest that the characteristic neuronal oscillatory activities emerged specifically during the CP may represent as neural activity trait markers for the experience-dependent maturation of developing visual cortical circuits.

Distinct neural oscillatory activities have been shown to support perceptual and cognitive functions in the mature brain^1^,^2^,^3^. For instance, synchronous oscillatory activities in the beta-gamma bands (20–90 Hz) were hypothesized to code the relationship among visual features^1^.These oscillations are known to be driven by the bottom-up visual inputs in the adult mammalians^4^,^5^ including the mice^6^, and can be modulated by top-down attention in monkeys^7^. During the brain development, neural oscillatory activities also play important roles in shaping the circuit organization and in establishing mature brain functions^8^,^9^,^10^. Diverse patterned synchronous oscillatory activities at early developmental stages in various brain areas have been observed, such as neuronal activity waves in the retina^11^, correlated bursts^12^ and gamma rhythms^13^ in the hippocampus, as well as delta brushes and spindle bursts in the neonatal cortex of humans and rodents^14^,^15^. These early oscillatory activities are important for the neural circuit development, which is best exemplified in the development of mammalian visual system. For instance, retina neuronal activity waves are known to instruct the binocular segregation in the dorsal lateral geniculate nucleus (dLGN)^16^ and the formation of precise retinotopic map in the primary visual cortex (V1)^17^, following a Hebbian synaptic learning rule^18^. During human childhood and adolescent periods, there are a decrease of low (delta, theta) frequency band oscillations and a simultaneous increase of higher (beta, gamma) band oscillations in the resting-state and task-related brain activities^8^,^19^,^20^,^21^. An aberrant development of these oscillatory activities has also been reported in neuropsychiatric patients^22^. While oscillatory activities in the early (perinatal and neonatal) and later developmental stages are relatively well characterized, little is known about how oscillatory activities change across a postnatal critical period (CP), during which the brain circuits are most malleable to environmental experience.

The postnatal CPs for experience-dependent brain plasticity exist in many sensory cortical circuits, such as primary visual^23^,^24^, auditory^25^ and somatosensory^26^ cortices, as well as those for the language^27^ and social interaction^28^. Based on the idea of the CP plasticity, the eye patching during the CP has widely been used to treat the amblyopia^29^ clinically in children, while other forms of training during the CP in early childhood greatly promote the development of cognitive functions, such as language learning^27^. Abnormal development related to this period has also been observed in some developmental psychiatric disorders like the autism and schizophrenia^8^,^22^. One of the best characterized forms of CP plasticity is the early visual inputs-dependent ocular dominance (OD) plasticity of the developing V1 in mammals including primates^30^, cats^23^, ferrets^31^ and rodents^24^. Accumulating studies in mice during the last decades provided much knowledge about the function of this V1 CP plasticity, such as promoting the developmental emergence of functional microcircuits^32^ and the binocular matching^33^, and its underlying molecular and cellular mechanisms^34^,^35^,^36^. However, the neural activity substrates for the plasticity during the CP were rarely studied^37^,^38^. Given the importance of patterned activity in mediating experience-dependent plasticity^39^, the synchronous oscillatory activities in the developing V1 may potentially play roles in the experience-dependent circuit maturation during the CP. However, whether and how specific neural oscillations in the mammalian V1 developmentally change across the CP remains elusive.

In the present study, we characterized the development of neural oscillatory activities across the CP up to the adult period, by performing recordings of local field potentials (LFPs) and single units in the V1 of head-fixed awake mice before (pre-CP, P17–19), during (P26–30) and after (P60–90) the CP. Our results demonstrate that from the pre-CP to CP, the peak frequency of spontaneous fast oscillations shifted from the beta band to the gamma band, accompanied with decreasing cross-frequency coupling (CFC) and broadband spike-field coherence (SFC), suggesting that distinct fast oscillatory activities emerge and gradually decouple from lower frequency bands with the maturation of V1. Moreover, visual stimulation markedly increased the beta band but decreased the gamma band activity from the CP onwards, but not in the pre-CP, suggesting that strong visually induced beta oscillation appears first during the CP in addition to the maturing of gamma oscillation during spontaneous activity. Further experiments using dark-reared mice suggest that the developmental emergence of beta and gamma band oscillations is dependent on visual experience after the eye opening. The initial appearance of adult-like neural oscillations during the CP corresponds well with the refinement^32^ and functional maturation^33^ of visual cortical circuits and can represent as a neural activity trait marker of the cortical maturation during this period.

## Results

We performed extracellular recording from the binocular zone of V1^40^,^24^ as well as from the dLGN in head-fixed awake mice at 3 different postnatal ages: P17–19 (n = 19 mice), P26–30 (n = 19), and P60–90 (n = 16) referred to as the periods before (pre-CP), during and after (Adult) the CP of experience-dependent OD plasticity, respectively^24^. In the experiments, full-screen sinusoidal drifting gratings (100% contrast) with 12 different orientations and spatial frequency of 0.02 or 0.04 cycle per degree (cpd) and temporal frequency of 3 Hz were used as visual stimuli. The significances of statistical difference (*p* values) were all tested with an unpaired *Kolmogorov–Smirnov* test.

### Developmental maturation of the spontaneous LFP power spectrum

**Figure 1:**
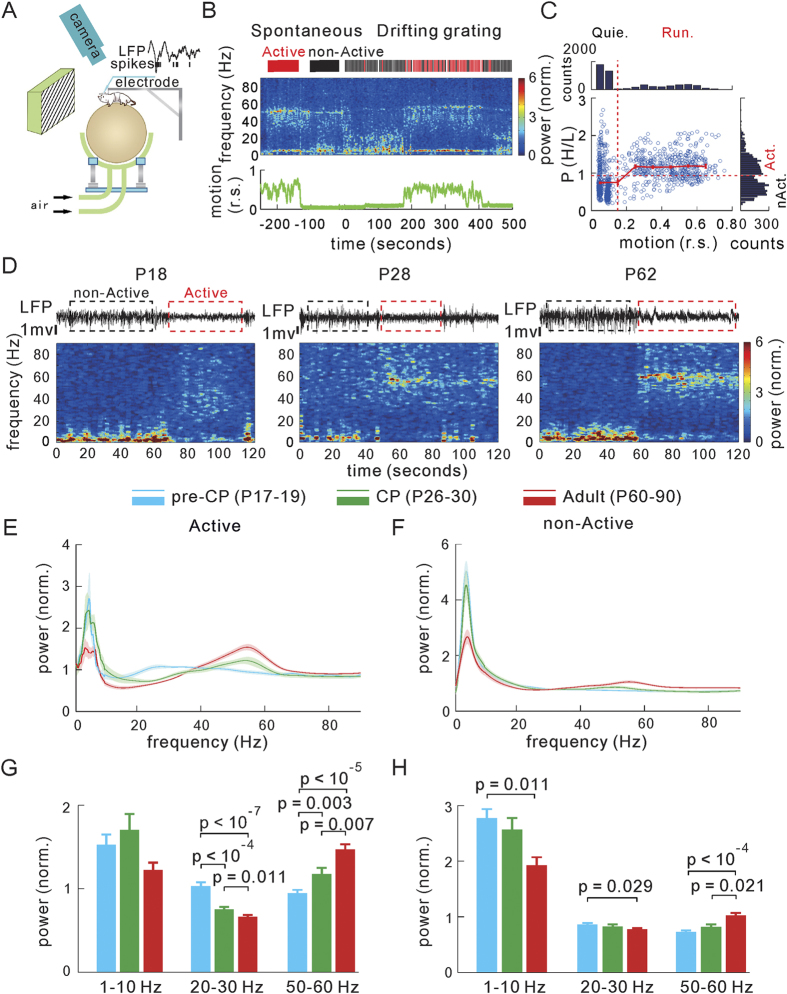
Developmental changes of spontaneous LFP’s power spectrum. (**A**) A diagram showing the experimental setups with visual stimulation, behavior monitoring and electrophysiological recording in the head-fixed awake mouse. The animal could stand still or run on a Styrofoam ball suspended on air. (**B**) Time-frequency power spectral analysis of the LFP recorded during the periods without (spontaneous) and with visual stimuli (drifting grating, top). The relative speed (r.s., arbitrary unit) of the animal was calculated from video scenes (25 frames/s, the relative speed of motion was frame-to-frame pixels’ luminance changes at 5 frames/s with a down sampling, bottom, details see Supplementary Fig. 1). The durations or stimulation trials of Active (Act.) and non-Active (nAct.) states are indicated by the red and black bars, respectively (top). (**C**) Correlation plot of the power ratio of high (H: 40–70 Hz) and low (L: 1–20 Hz) frequency bands, P(H/L), to the animal motion (r.s.). Each point represents the mean values of 1-s windows from the continuous recording shown in (**B**). Solid red line: averaged values of P(H/L) calculated with a bin width of 0.1 r.s. Dash red line: vertical line indicates the r.s. value (0.15) to classify Quiescent (Quie.) and Running (Run.) states, horizontal line indicates the mean P(H/L) of all data points. Inserts: histogram distributions of the P(H/L) (right) and the relative speed (top), respectively. (**D**) Example spontaneous LFPs (top) and their power spectrogram in the non-Active (black box) and Active (red box) states in the mice at P18 (pre-CP), P28 (CP) and P62 (adult). (**E,F**) Averaged power spectrum of all examined mice at three stages (pre-CP: P17–19, n = 16 mice; CP: P26–30, n = 13; adult: P60–90, n = 13) in the Active (**E**) and non-Active (**F**) states. Shadow areas indicate the mouse-to-mouse s.e.m. (**G,H**) Changes of power of delta/theta (1–10 Hz), beta (20–30 Hz) and gamma (50–60 Hz) band activities in the Active (**G**) and non-Active (**H**) states among the three stages. Error bars represent mouse-to-mouse s.e.m. The *p* values were calculated by the unpaired *Kolmogorov–Smirnov* test.

Our experimental setup, modified from the one by Dombeck *et al.* (2007)^41^ and Niell *et al.* (2010)^42^, is shown in Fig. 1A. Mouse’s head was fixed to a crossbar, so that the animal could either stand still or run freely on a Styrofoam ball floated on a stream of air. A custom-made electrode array (4–6 channels) was placed in V1 to record LFPs and spikes. A video camera was placed above the animal to record its behaviors in part of the experiments, and a relative speed (r.s., in arbitrary unit) of animal behavior was estimated based on the pixels’ luminance intensity changes of consecutive video frames (Supplementary Fig. 1A). Visual stimulus was presented through a cathode-ray tube (CRT) monitor, which was placed about 20 cm away in front of the mouse, covering about 90^o^ × 75^o^ of the visual field as described in our previous study^38^ (Fig. 1A). A power spectrogram of the LFP recorded without visual stimulus (defined as spontaneous activity) and with visual stimulus (induced activity), is shown in Fig. 1B. The corresponding relative motion of the animal is shown in green at the bottom. The LFP power spectrogram commonly was composed of alternating two states, one of which had larger power in the high frequency band (40–70 Hz) and smaller power in the low frequency band (1–20 Hz) than the other (Fig. 1B top). This LFP state transition depended on the animal’s motion^42^, where also two behavior states could be distinguished and defined as: the period with r.s. <0.15 was considered as the quiescent state while that with r.s. >0.15 was regarded as the running state of the animals (Fig. 1C top). The LFP power in the high frequency band [P (H: 40–70 Hz)] during running periods was on average much larger than that during quiescent periods, and the power in low frequency band [P (L: 1–20 Hz)] was oppositely changed (Supplementary Fig. 1B,C). To quantitatively distinguish periods of the active and non-active local brain states from the LFP itself, the ratio of high (H) and low (L) frequency band power [P(H/L)] was used: active and non-active states were defined as in which the mean P(H/L) was significantly larger and smaller than the global mean power ratio in each recording, respectively (transitions between the two states existed in near all of our recordings, Fig. 1C). This definition of active and non-active LFP states basically correlated with the defined running and quiescence behavioral states (Fig. 1C). Thereafter the two brain states are defined primarily on the basis of the above LFP analysis in the following experiments (see Methods for the procedure details).

We then examined developmental changes of the LFP’s power spectrum in the V1 before, during and after the CP (Fig. 1D). By quantifying the normalized relative LFP power for both active and non-active brain states separately (see Methods), we found that in the active state, the relative power of the beta frequency (20–30 Hz) band markedly decreased from the pre-CP to the CP onwards (pre-CP: 1.03 ± 0.05, n = 16 mice; CP: 0.76 ± 0.03, n = 13; adult: 0.67 ± 0.02, n = 13), while the power in gamma frequency band (mean values in 50–60 Hz) displayed an increasing trend over the same periods (pre-CP: 0.95 ± 0.04; CP: 1.18 ± 0.08; Adult: 1.47 ± 0.06; Fig. 1E,G). In the non-active state, this trend of the developmental change of LFPs was preserved, albeit with smaller values and difference between ages (Fig. 1F,H). Mean power of the lower frequency range in the delta/theta bands (1–10 Hz) slightly decreased from the pre-CP (2.77 ± 0.16) to the adult (1.93 ± 0.14) only in the non-active state (Fig. 1H), but not in the active state (Fig. 1G). Thus our results demonstrate an increased power in the gamma band (40–65 Hz) and a reduced power in lower frequency range (most significant in the beta band, 20–30 Hz) during spontaneous activity mainly in the transition from pre-CP to CP. The peak frequencies and frequency ranges of the increased gamma band power bumps (in CP and adult ages) showed relatively small variability among examined mice (Supplementary Fig. 3). Mean values of the peak frequencies were 52 ± 4.4 (n = 13 mice; mean ± s.d.) and 54 ± 2.1 Hz (n = 13), for mice at the CP and adult stages, respectively, while the corresponding mean values of the half peak frequency widths were 18.9 ± 8.7 and 21.8 ± 5.0 Hz. We also conducted multiple sites recording of electric activities from the V1, the bilateral neck muscles and the agarose above the cortical surface simultaneously in two adult mice to rule out the possible artifacts of the increased gamma band (40–65 Hz) activity due to the muscle activity, line noise (50 Hz) and other unknown interference during the recording, especially during the active (running) state (see Methods). The results indicate that the cortical gamma band activity is not artifact of the muscle activity and other unexpected electrical noise in our recording setup because there were no similar gamma band power bump in muscle and noise activities and no gamma band coherence spectrum^43^ bump between LFP and muscle or noise activities (Supplementary Fig. 2).

We further observed that the developmental power increase of spontaneous gamma band activity did not vary with cortical depths or layers neither when based on the estimated depths of the custom-made electrode recording sites (spanning from 300 μm to 800 μm and mainly corresponding to cortical layers 4–5^38^) nor when based on the recordings sites of the linear silicon electrodes (A 1 × 16 or 32 probe, Neuronexus Tech. Inc.) in adult mice (Supplementary Fig. 4). Our subsequent recording from the dLGN also implicated a similar developmental change in the power spectrum of spontaneous LFPs (Supplementary Fig. 5). These results suggest that the developmental change of spontaneous neural oscillations in the V1 may depend on the maturing of the thalamocortical inputs.

### Developmental decrease of spontaneous CFC and SFC

**Figure 2:**
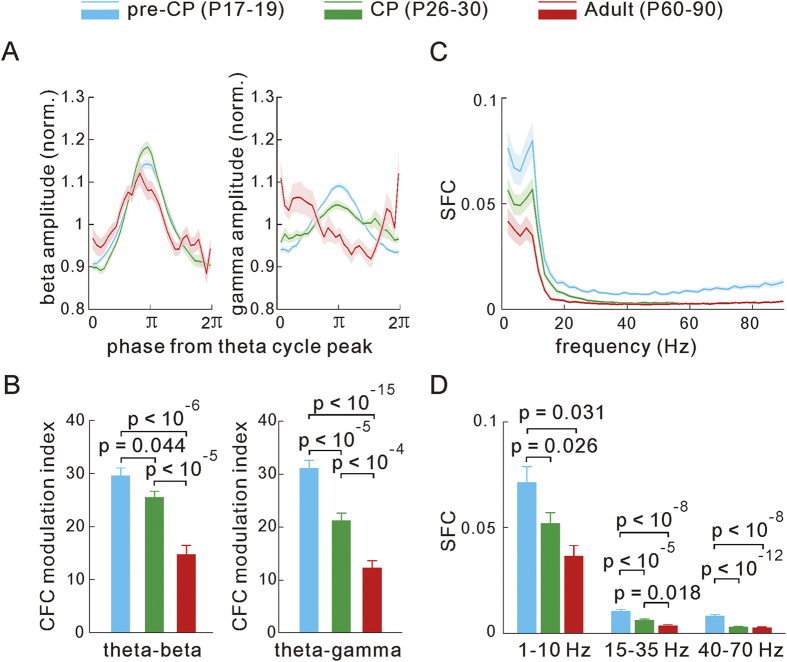
Developmental changes in the cross-frequency coupling (CFC) and spike-field coherence (SFC) of spontaneous activity. (**A**) The normalized LFP amplitudes at beta (15–35 Hz, left) and gamma (40–70 Hz, right) bands corresponding to the phase of theta (3–8 Hz) cycles in the pre-CP (n = 135 recordings in 19 mice), CP (n = 150 in 17 mice) and adult (n = 70 in 15 mice) mice. The data in the active and non-active states are pooled (states pooled). (**B**) Summarized results of the phase-amplitude CFC modulation index in mice at different stages. (**C**) The average SFC in the frequency range of 1–90 Hz for all recorded single units from mice at three stages (pre-CP, n = 77 units in 19 mice; CP, n = 106 in 16 mice; adult, n = 35 in 15 mice; states pooled). (**D**) Quantification of SFC of (**C**) in three frequency bands (1–10 Hz, 15–35 Hz, 40–70 Hz). Shadow areas in (**A**,**C**) represent the site-to-site and the unit-to-unit s.e.m., respectively. Error bars in (**B**,**D**) represent s.e.m. The *p* values were calculated by the unpaired *Kolmogorov–Smirnov* test.

Power spectral analysis reflects only the amplitude of each frequency band^44^, however, the coupling strength between different LFP frequency bands (cross-frequency coupling, CFC) and the spike-field coherence (SFC) are two more indexes for the integration level of population neural activities^45^,^46^,^47^. Changes of the CFC and SFC have been observed in response to perceptual or cognitive tasks^7^,^48^,^49^,^50^, and in the aged or diseased brain^50^. In this study, we also examined the development of the CFC and SFC during spontaneous activity in mouse V1. We calculated the theta (3–8 Hz) to beta (15–35 Hz) and gamma (40–70 Hz) bands phase-amplitude CFC in the active and non-active states during pre-CP, CP and adult ages (see Methods). The reason for why we chose these particular 40–70 Hz range in the gamma band is because of the relative stable power bumps in this range among examined mice at the CP and adult ages (Supplementary Fig. 3). We found that the peak power of both the beta and gamma bands was preferentially phase-locked to the trough (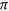) of theta band cycles in both active and non-active states (states pooled: data pooled from both states; Fig. 2A). Quantification of the phase-amplitude modulation index (see Methods), which reflects the CFC strength^51^, further suggests a developmental decrease of theta-beta and theta-gamma band CFC in mice from pre-CP to the CP onwards (pre-CP, theta-beta: 29.6 ± 1.4, theta-gamma: 31.0 ± 1.5, n = 135 recording channels in 19 mice; CP, theta-beta: 25.5 ± 1.2, theta-gamma: 21.2 ± 1.4, n = 150 in 17 mice; adult, theta-beta: 14.7 ± 1.8, theta-gamma: 12.3 ± 1.3, n = 70 in 15 mice; states pooled; Fig. 2B). The developmental changes of the CFC during active and non-active states were similar although both the theta-beta and theta-gamma band CFC strengths were about twice larger in non-active state than in active state (Supplementary Fig. 6A,B).

We further calculated the SFC, a measure of synchronization between spikes and field potential^47^,^48^, following a method described in a previous study^48^. First, we averaged LFP segments around each spike (spike-triggered average: STA) with a time window of ±200 ms during spontaneous activity. We then measured the spike-triggered power spectrum by averaging the power spectra of each of the LFP segments. The SFC was then calculated as the ratio of the power spectrum of the STA over the spike-triggered power spectrum (see Methods and Supplementary Fig. 8D,E for procedure details). The SFC in a broadband frequency range (1–90 Hz) for all putative single units recorded from the V1 is shown in Fig. 2C (states pooled). In agreement with the larger CFC strength in the non-active state, we first observed considerably larger values of SFC (measured at 3 frequency ranges) in the non-active state than the active state (Supplementary Fig. 6C,D). Second, there was a similar global reduction of SFC values from the pre-CP to the CP onwards (pre-CP, n = 77 units in 19 mice; CP, n = 106 in 16 mice; adult, n = 35 in 15 mice; states pooled; Fig. 2D) regardless of the states (Supplementary Fig. 6C,D), except that there was no significant difference in the delta/theta band (1–10 Hz) between pre-CP and CP only during non-active state (Supplementary Fig. 6D). Thus, the decrease of both the CFC and the SFC across a broad frequency range with the development of V1, in particular during the transition from the pre-CP to CP, suggests a developmental de-correlation of the spontaneous population activity.

### Developmental change of the visually induced beta and gamma band LFP activities

**Figure 3:**
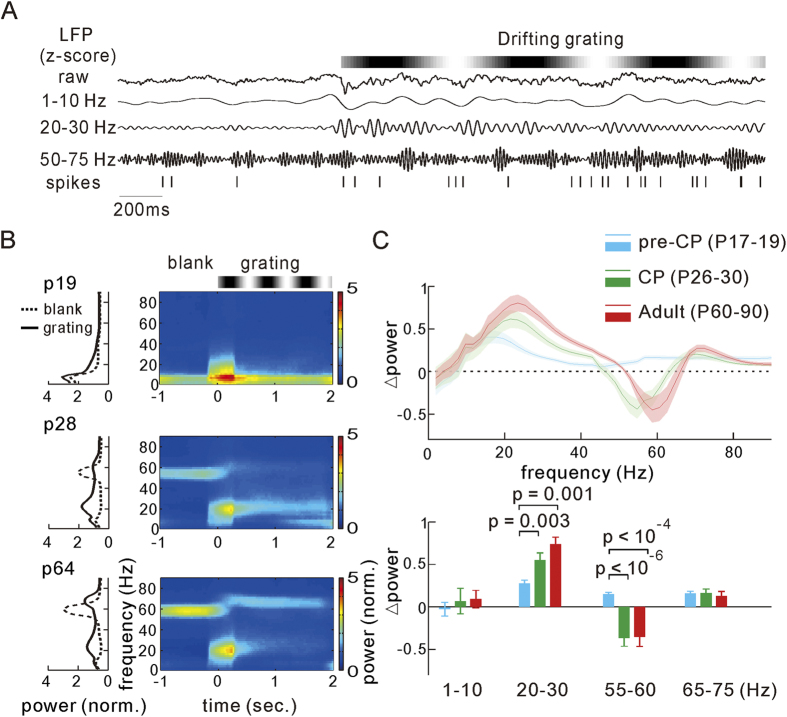
Development changes of visually induced LFP’s power spectrum. (**A**) Traces of raw and filtered LFPs at different bands, as well as recorded spikes measured before and during the drifting grating stimulation (indicated by the grating bar). (**B**) The spectrograms (right) and the corresponding plots of the averaged power (over the time) along with the frequencies (left) during the blank and visual stimulation periods in mice at the pre-CP, CP and adult stages (states pooled). (**C**) Visually induced power change of LFPs (∆power: grating-blank) in the 1–90 Hz range (top) and the average values of ∆power at discrete frequency bands (bottom) in mice at the 3 stages (pre-CP, n = 19 mice; CP, n = 16; adult, n = 19; states pooled). Shadow areas and error bars represent the mouse-to-mouse s.e.m. The *p* values were calculated by the unpaired *Kolmogorov–Smirnov* test.

We further asked whether visually induced oscillatory activities in the V1 were also developmentally modulated across the CP. In this set of experiments, drifting gratings were presented to awake mice either in the active state or non-active state, which were characterized by the analysis of P(H/L) (Fig. 1B,C and see Methods). As there are no clear orientation columnar structures in the mouse V1^52^, we pooled the induced LFP or spike activity across gratings of all different orientations together in the subsequent data analysis. An example trial of the LFP traces (raw and band-pass filtered) and the simultaneously recorded spike train are shown in Fig. 3A. Then, we conducted a time-frequency spectral analysis (Fig. 3B right) and computed their time-averaged power spectrum (Fig. 3B left) of the LFPs recorded during the baseline period (blank) and during the visual stimulation in mice at the pre-CP, CP and adult stages, separately (states pooled, Fig. 3B). We found that the drifting gratings generally induced an elevation of beta band (in the range of 15–35 Hz with peak at ~22 Hz) but a suppression of the gamma band (with a dip peak in the range of 55–60 Hz) power in the mice during CP and adulthood (Fig. 3B middle and bottom). Moreover, we observed a shift of the peak frequency of the gamma band to a slightly higher frequency (~70 Hz) when compared to spontaneous activity during the blank period (Fig. 3B middle and bottom). However, visual stimulation affected the oscillatory activities much less during the pre-CP (Fig. 3B top). Furthermore, the population change of LFP power spectrum measured before (blank) and during the visual stimulation (∆power: grating-blank) further showed that the power change in the beta band activity (measured over 20–30 Hz) during the CP (n = 16 mice) and adult (n = 19) was substantially higher than that during pre-CP (n = 19), both in the active and non-active states (states pooled, Fig. 3C and Supplementary Fig. 9A,B). The visually induced suppression of the spontaneous gamma band (in the range of 50–65 Hz, mean values from the 55–60 Hz range were compared) activity was also significantly larger during CP and adult period (states pooled, Fig. 3C), especially in the active state (Supplementary Fig. 9A,B).

On contrast, the power in the delta/theta (1–10 Hz) band showed differential visual induced changes in the two states: a slight increase in the active state but a strong decrease in the non-active state consistently in most examined mice at three different stages (Supplementary Fig. 7A–F and Supplementary Fig. 9A,B), which led to no significant visual induced changes of power in this band at all three stages when the data were pooled from the two states (Fig. 3C). We noted that there were some exceptional recordings from few mice in which the induced power in the delta/theta (1–10 Hz) band was increased and the theta band activity was reset by the stimulus onset in the non-active state (Supplementary Fig. 7C,F). Our further analysis of the power spectrum of the stimulation-trial averaged LFPs suggested that stimuli-induced change of the delta/theta band activity might be attributed to a state-dependent modulation of endogenous oscillations rather than a potential phase entrainment^53^ by the stimuli’s temporal frequency of 3 Hz (Supplementary Fig. 7D–F). Along with this visually induced low frequency band power change the theta-beta and theta-gamma CFC also exhibited a similar trend of visually induced changes (Supplementary Fig. 7G–I). However, the visual modulation to the low frequency band power and the related CFC has little developmental difference among the three stages (Supplementary Fig. 7).

Consistently, subsequent investigation of the strength of oscillatory activity with depths or layers [the current source density (CSD) analysis^54^] based on recording of induced LFPs with our custom-made or linear silicon electrodes in the V1, respectively (Supplementary Fig. 4) and additional recordings in the dLGN (Supplementary Fig. 5) further suggested that the induced beta and gamma band neural oscillations might mainly originate from the layer 4 circuit of the mouse V1.

### Developmental modulation of the visually induced SFC in beta and gamma frequency bands

**Figure 4:**
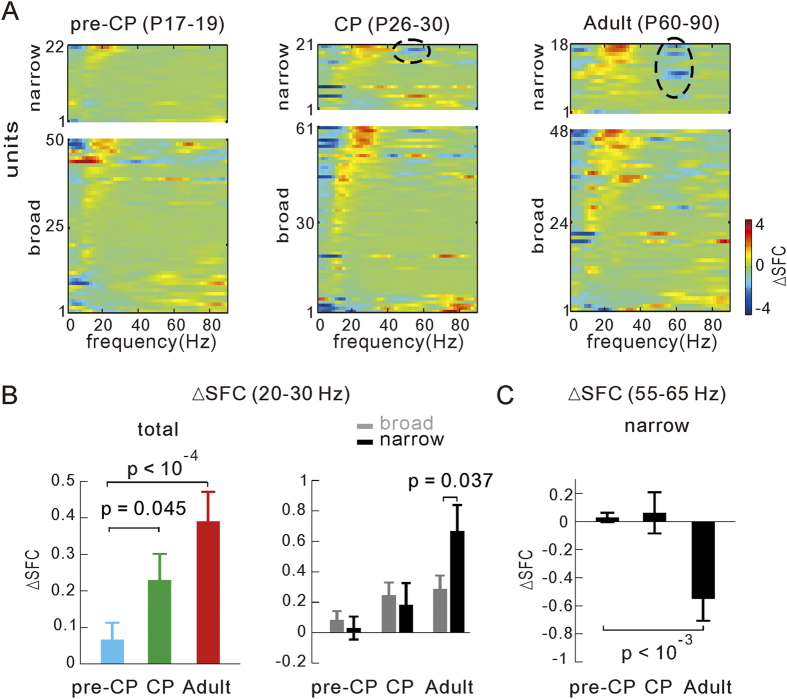
The visually induced changes of SFC over the development. (**A**) Visually induced ∆SFC (grating-blank, z-score) in a range of 1–90 Hz. The broad- and narrow-spiking units recorded in mice at three ages (pre-CP, n = 72 units in 17 mice; CP, n = 82 units in 14 mice; adult, n = 66 units in 15 mice; states pooled). The plots of each unit are sorted based on its ∆SFC value at the beta band (20–30 Hz). Black circles indicate that visually induced decreased SFC at the gamma band mainly occurs to the narrow-spiking units. (**B**) Summarized results of average ∆SFC at the beta band (20–30 Hz) for total units (left) or separated broad- and narrow-spiking units (right) in mice at 3 stages. (**C**) Summarized results of average ∆SFC at the gamma band (55–65 Hz) for narrow-spiking units in mice at 3 stages. Error bars represent s.e.m. The *p* values were calculated by the unpaired *Kolmogorov–Smirnov* test.

In addition to the LFP power spectra, we further analyzed effect of visual stimulation on the SFC. Higher values of the SFC reflect more synchronous oscillatory firing of local population neurons^47^,^48^. We obtained the STA and subsequently the SFC for neuronal activities recorded during the blank and the visual stimulation periods, respectively (see Methods and Supplementary Fig. 8D,E). The difference [∆SFC (grating-blank)] of the SFC (z-score) during the visual stimulation and blank periods showed a remarkable visually induced increase in the beta band and a decrease in the gamma band coherence between the single unit and the LFP in the example recording (Supplementary Fig. 8F). The pooled results of ∆SFC across all recorded single units (mean firing rate > 1 Hz, evoked peak rate > baseline rate + 3 × s.d.) are plotted in Fig. 4A (states pooled). Based on the spike waveforms of extracellularly recorded neuronal units, we further categorized units into broad- and narrow-spiking to separate putative excitatory principal cells and inhibitory cells^42^,^55^, respectively (see the Methods and Supplementary Fig. 8A–C). The mean ∆SFC of all units (states pooled, Fig. 4B left) in the beta band (20–30 Hz) increased significantly from the pre-CP (n = 72 units in 17 mice) to the CP (n = 82 in 14 mice, p = 0.045, *vs* pre-CP) and adult (n = 66 in 15 mice, p < 10^−4^, *vs* pre-CP). This developmental change was stronger in the active state (CP *vs* pre-CP: p < 10^−3^; adult *vs* pre-CP: p < 10^−3^; Supplementary Fig. 9C) than in the non-active state, in which only difference existed between the pre-CP and adult (p = 0.002, Supplementary Fig. 9D). Interestingly, the beta band mean ∆SFC of narrow-spiking units appeared to have even larger developmental increase than that of broad-spiking units, especially in the adult stage (states pooled: p = 0.037; active state: p = 0.003 ; non-active state: p = 0.034; narrow *vs* broad; Fig. 4B right and Supplementary Fig. 9C,D). We also noted a significant visually induced reduction of the gamma band mean SFC for some narrow-spiking units (indicated by black circles in Fig. 4A and Supplementary Fig. 8E,F) during the CP (n = 21 units, states pooled: ns; active state: p < 10^−5^; non-active state: ns; *vs* pre-CP) and adulthood (n = 18 units, states pooled: p < 10^−3^; active state: p < 10^−5^; non-active state: p = 0.012; *vs* pre-CP), but not during pre-CP (n = 22 units, Fig. 4C and Supplementary Fig. 9C,D). These developmental changes of ∆SFC are consistent with our above observation of the visually induced LFP power spectrum change over the development (Fig. 3). Relative larger changes in the beta and gamma band ∆SFC of narrow-spiking units may imply that the activity of putative cortical inhibitory cells might exert more important contribution than excitatory neurons to the developmental maturing of neural oscillations in the mouse V1.

### Correlated changes of firing rate, transmission strength and tuning property

**Figure 5:**
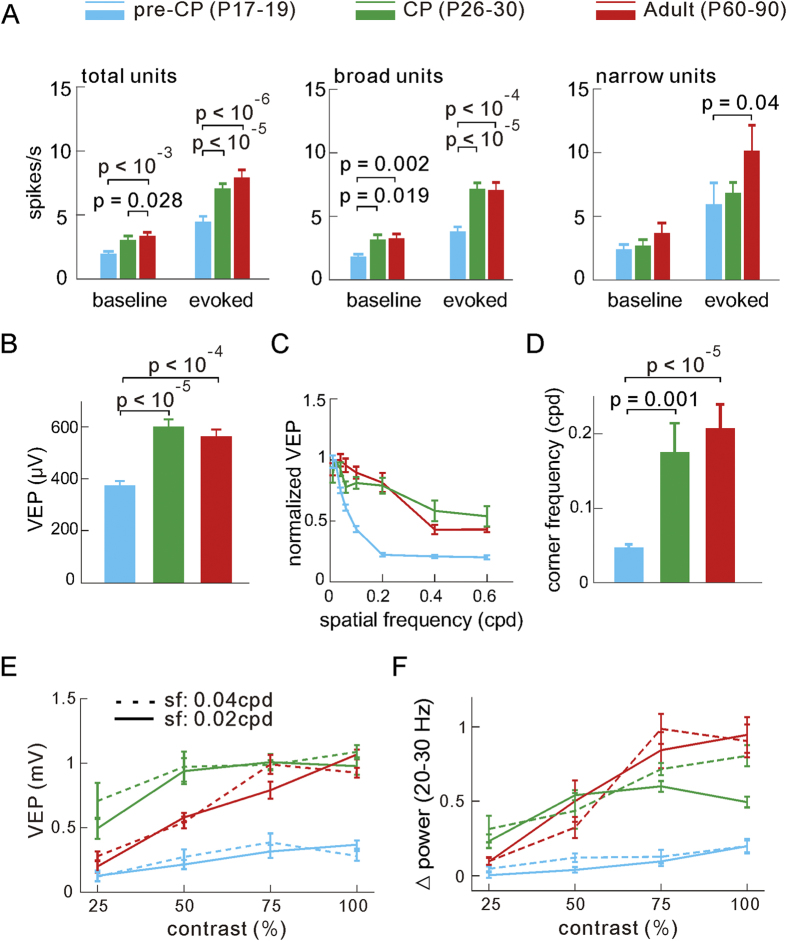
Correlated changes of firing rate, visual evoked potentials (VEPs) and spatial frequency tuning. (**A**) Developmental changes of the spike rate measured before and during the grating stimulation. The rate of the baseline and evoked spikes were measured in a 2 s window before the stimulus and in a 100 ms window after the stimulus onset, respectively, and averaged among all units (left), broad- (middle) and narrow- (right) spiking units. The number of recorded units in the 3 stages are: for broad units: pre-CP, n = 77 in 17 mice; CP, n = 82 in 14 mice; adult, n = 52 in 15 mice; for narrow units: pre-CP, n = 29; CP, n = 29; adult, n = 20. (**B**) Change of VEP amplitudes (recorded in layers 4–5) at three stages (pre-CP, n = 101 recording sites in 17 mice; CP n = 87 in 14 mice; adult, n = 107 in 15 mice). (**C**) Change of the spatial frequency tuning characterized by VEPs (pre-CP, n = 30 in 6 mice; CP, n = 24 in 4 mice; adult, n = 17 in 5 mice). (**D**) Change of the corner frequency. (**E,F**) Plots of VEP amplitude (**E**) or visually induced beta band power (**F**) *vs* the contrast levels of grating stimulus at the 0.02 and 0.04 cpd spatial frequencies in mice at the 3 stages. Error bars represent s.e.m. The *p* values were calculated by the unpaired *Kolmogorov–Smirnov* test.

Because visual input strength^56^ and neuronal firing rate^57^ might influence neuronal oscillations, we next examined whether these two factors are developmentally modulated. We first compared the mean firing rate during baseline (blank) and evoked activity among the three stages, and found that firing rate was increased mainly in the broad-spiking units from the pre-CP (77 broad and 29 narrow units in 17 mice) to the CP (82 broad and 29 narrow units in 14 mice) and adult (52 broad and 20 narrow units in 15 mice; Fig. 5A). Second, peak amplitudes of the visual evoked potentials (VEPs) in response to drifting grating stimuli (12 orientations at the spatial frequency of 0.02 or 0.04 cpd and at the temporal frequency of 3 Hz), recorded mainly from layers 4–5, also significantly increased from the pre-CP (n = 101 recording sites in 17 mice) to the CP (n = 87 in 14 mice; p < 10^−5^, *vs* pre-CP) and adult (n = 107 in 15 mice; p < 10^−4^, *vs* pre-CP; Fig. 5B), suggesting a developmental strengthening of thalamocortical inputs^58^,^59^. The above increase of the spike rate and VEPs in V1 could in principle also be attributed to an altered tuning of the developing V1 cells to the visual inputs, in particular a changed spatial frequency tuning, which is known to undergo a significant change from the pre-CP to the CP^60^. We thus also compared the spatial frequency tuning in the three developmental stages (Fig. 5C). The preferred spatial frequency, estimated from the VEP amplitudes in response to phase reverse static grating stimuli, differed little among the pre-CP, CP and adult stages (~0.02 cpd, Fig. 5C). Note that we used this preferred value for inducing neural oscillatory activities in most above experiments. The corner (cutoff) frequency, defined as the frequency where the VEP amplitude dropped to 0.707 of the peak, increased from 0.047 ± 0.005 in pre-CP to 0.175 ± 0.039 in CP and 0.207 ± 0.032 cpd in adult (Fig. 5D). This finding that higher spatial frequencies evoked increased responses in CP and adult is consistent with the previous finding of enhanced visual acuity during the mouse vision development^60^. Since the optimal spatial frequency in V1 was almost constant, we can exclude the possibility that the observed changes in oscillatory activity was only due to a changed visual input preference of V1 at different stages. Moreover, consistent with other studies^56^,^59^, the VEP amplitudes and the power of induced beta band activity were proportional to the contrast of drifting gratings (at the spatial frequency of 0.02 or 0.04 cpd) in the 3 stages (Fig. 5E,F). Taken together, our results indicate that the increase of visual input strength during the development may in part account for the enhancement of visually induced beta oscillation in the developing V1 from the pre-CP to the CP.

### Dark rearing retards the emergence of adult-like neural oscillatory activities

**Figure 6:**
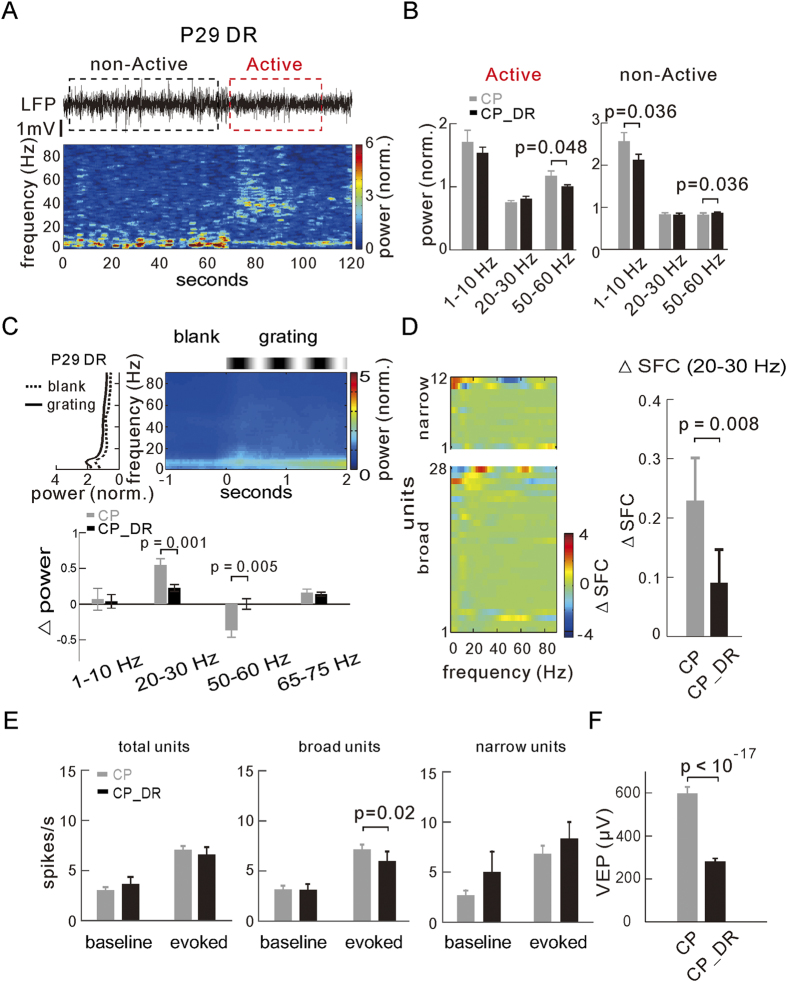
Effects of dark rearing on the maturation of neural oscillations. (**A**) Example spontaneous LFPs (top) and their power spectrogram in the non-active (black box) and active (red box) states in the dark reared mice at P29 (CP_DR). (**B**) Comparison of average power of spontaneous activities at the delta/theta (1–10 Hz), beta (20–30 Hz) and gamma (50–60 Hz) bands during the active (left) and non-active (right) states between the dark-reared (n = 13 mice) and normal-reared mice at the CP (P26–30). (**C**) Top, the power spectrogram (right) and the corresponding plot of the averaged power (over the time) along the frequencies (left) during the blank and visual stimulation periods in dark-reared mice (scale bar is the same as the normal-reared mice for comparison). Bottom, difference of the ∆power at discrete frequency bands between the dark-reared (n = 12 mice) and normal-reared mice (states pooled). (**D**) Visually induced ∆SFC for the broad- and narrow-spiking units (left) and the average ∆SFC at the beta band (20–30 Hz) for all units recorded in dark-reared mice (n = 40 units in 12 mice, right, states pooled). (**E**) The baseline and evoked spike rates of all units (left), broad- (middle, n = 39) and narrow- (right, n = 15) spiking units recorded in dark-reared and normal-reared mice. (**F**) Comparison of VEP amplitudes between the dark-reared (n = 169 recording sites in 12 mice) and normal-reared mice at CP. Error bars represent s.e.m. The significance (*p* values) of difference between data groups is tested by the unpaired *Kolmogorov–Smirnov* test.

Deprivation of early visual experience during development, e.g. dark rearing, has shown to delay the maturation of V1 and the timing of the onset of the CP^61^,^62^,^63^. We thus also examined whether the observed change in neural oscillatory activity during development was dependent on the early visual experience. For that, mice were reared in total darkness from the birth till the day of recording (P28–30, normal CP), and the spontaneous and visually induced neural activities of V1 were recorded and analyzed. We found that, in comparison with the normally reared mice (Figs 1, 2, 3, 4, 5), dark-reared mice showed smaller power in the spontaneous gamma band during active state (n = 13 dark-reared mice, p = 0.048; Fig. 6A,B) and different peak frequency around 40 Hz instead of that (20–40 and 52 ± 4.4 Hz) for normal mice in the pre-CP and CP, respectively (Fig. 6A). On the other hand, in the non-active state, delta/theta band power was slightly reduced in dark-reared mice, whereas gamma band power increased slightly (delta/theta: p = 0.036; gamma: p = 0.036, *vs* normal CP mice; Fig. 6B right). Strikingly, the increase of beta band power was absent in dark-reared mice when visually stimulated in both active and non-active states (n = 12 mice, states pooled: p = 0.001; active state: p = 0.035; non-active state: p = 0.013), and the visually induced suppression of gamma band power was also weaker than in normally reared mice (states pooled: p = 0.005; active state: p < 0.001; non-active state: ns; Fig. 6C and Supplementary Fig. 9E,F). Similarly, in contrast to normally reared mice, the visually induced SFC was not increased in the beta band in dark-reared mice (states pooled: p = 0.008; active state: p < 10^−3^; non-active state: p = 0.007; Fig. 6D and Supplementary Fig. 9G,H). We also found that dark rearing slightly reduced the evoked fire rate of broad-spiking units (n = 39, p = 0.02) but not that of the narrow-spiking units (n = 15, Fig. 6E), along with a significant decrease of the VEP amplitude by about 50% (n = 169 recording sites in 12 dark-reared mice, p < 10^−17^, *vs* normal CP mice; Fig. 6F). Taken together, deprivation of early visual experience affected the development of oscillatory activities in V1, potentially caused in part by the delayed strengthening of thalamocortical inputs in dark-reared mice.

## Discussion

The present study provides a systematic characterization of the developmental evolution of neural oscillatory activities in the V1 of awake mice across the CP up to the adulthood period. The results suggest that gamma band oscillations emerge and become dominant in spontaneous fast oscillatory activities specifically from the pre-CP to CP, accompanied by a gradual developmental decrease of CFC and SFC. On contrary, full-field drifting grating stimulation increases the beta-band but reduces the gamma-band LFP activity from the CP onwards, accompanied by the visually induced increase in the beta band but a decrease in the gamma band spike-field coherence. These developmental changes of neural oscillations can be retarded by the dark rearing treatment, indicating its dependence on early visual experience. Such developmental switch of characteristic spontaneous and stimuli-induced beta and gamma oscillations during the CP may present as neural activity trait markers for maturation of visual cortical circuits and involve in the visual processing and plasticity during this period. This development of oscillatory activities in the mouse cortex is reminiscent of the previous findings that EEG oscillatory patterns also change during the human brain development^19^. Patients of development-related neuropsychiatric diseases show abnormal EEG activities^8^,^22^. Thus, our systematic characterization of the development of cortical oscillatory activities across the CP could provide a new ground for a better understanding of neural activity evolution of the developing brain in health and disease.

Patterns of the spontaneous activity in the resting state brain has been proposed to reflect the default functional connections^64^,^65^. Accordingly, the strength of beta and gamma band oscillations is known to depend on connectivity patterns of local circuits^66^,^67^. In the present study, our LFP measurements from the developing V1 in awake mice demonstrate two distinct changes in the LFP power spectrum from the pre-CP to the CP: an emerged increase of power at the gamma (40–65 Hz) band but a reduction at the beta (20–35 Hz) band in both active and non-active brain states. These differential power spectrum changes at beta and gamma bands were accompanied by a gradual decrease in both the magnitudes of theta-beta and theta-gamma CFC and the broadband SFC from the pre-CP to adulthood. The latter CFC and SFC changes may suggest a continuous reduction of the correlation level of spontaneous population neuronal activities^50^ over the development of V1. This notion has also been supported by previous Ca^2 + ^imaging studies, in which a desynchronization^68^ or sparsification^69^ of spontaneous V1 population activity was observed during the period from the eye-opening to the CP. Given the deterministic role of local connectivity in the generation of these fast oscillations^66^,^67^, the differential evolution of spontaneous gamma and beta band oscillations across the CP of developing V1 may reflect the changing connectivity during the CP.

How visual inputs modulate neural oscillatory activities in the mature V1 has been extensively characterized in the cat^4^, monkey^5^ and mouse^6^,^42^,^56^. In the present study, we also provide a systematic view on how the induced oscillations mature over the mouse V1 development. First, our results suggest that it is from the pre-CP to the CP that there was a large increase of visually induced LFP beta band (15–35 Hz) activity in the developing V1, differed very little from that recorded from the adult stage, when awake mice were stimulated with drifting gratings. Correspondingly, we also observed a much larger increase of visually induced SFC in the beta band from the pre-CP to the CP, in comparison with that afterward the CP, especially in the active brain states. This 15–35 Hz range of induced beta oscillations during the CP and onwards is largely consistent with that found in previous studies, the 20–30 Hz range in anesthetized^56^ and awake^42^ mice, but slightly lower than the 25–50 Hz one found by another study in anesthetized mice^6^. In the V1 of cats under the anesthesia or in awaking states, visual inputs often elicited oscillations in a much higher frequency band (40–70 Hz)^4^,^48^. These results suggest that visual inputs consistently induce an increased cortical beta-gamma band oscillation in the developing (during CP) and mature V1 of the mice and other mammals. Second, we observed a substantial suppression of cortical baseline gamma band (55–65 Hz) activity in the CP and adult stages of awake animals, which is different from the previous observations of the increased cortical gamma band (40–70 Hz) oscillations in the awake^42^ and anesthetized^6^ mice, or in the cat under the both conditions^4^,^48^. It has been reported that the modulation of cortical gamma activity by visual stimuli is dependent on the behavioral state^42^ and the stimulation properties, such as the location^70^, size^71^, and orientation^4^. We note that in our recordings, there was a significantly larger baseline oscillatory activity in the gamma band in the CP and adult V1, especially during active state, in comparison with these previous studies in which no apparent power bumps in the gamma band were observed in the baseline LFP power spectrum^4^,^6^,^56^. Plus, we used full-field drifting gratings, which covered a large visual field (~90 degrees) of the mice, and the resultant surround inhibition^71^ could substantially modulate visual responses driven by the input from the central receptive field of recorded V1 region. We suspected that our observation of the visually induced suppression of gamma band activity may be attributed to these above factors in part. The dependence on the stimuli properties was further supported by the experiments using smaller size of drifting gratings, covering ~30 degrees visual field (close to the size of LFP’s receptive field of the recording site), or single moving bar (4 degrees width), and we found that these stimuli appeared to enhance the gamma but not much of the beta band oscillations in the similar recording conditions (Supplementary Fig. 10). Despite the difference in some oscillation properties between the previous studies and ours, our recording from awake mice at three different developmental stages has clearly demonstrated a maturation of the visually induced oscillations in beta and gamma bands in the developing V1 from the pre-CP to the CP.

Our results also showed that unlike the beta/gamma band activity, the baseline or visually induced lower frequency delta/theta band (1–10 Hz) activity underwent far less developmental changes across the CP in general. However, visual modulation of the lower band activity displayed much stronger dependence on the brain states. The LFP power at lower band and the corresponding coupling (CFC) with higher band activity were only slightly increased in the active state, but substantially decreased in the non-active state during the visual stimulation. This visual modulation on lower band activity appeared not to result from a potential phase entrainment^53^,^72^ to the temporal frequency (3 Hz) of drifting gratings (Supplementary Fig. 7). Thus, we conclude that the CP specific maturation of cortical oscillations in developing mouse V1 is confined more to the relatively higher frequency band (20–80 Hz).

The different contributions of excitatory-inhibitory (E-I) and inhibitory-inhibitory (I-I) synaptic circuits to the generation of neural oscillations have been extensively studied in the computational modeling^73^,^74^,^75^ and the experimental studies^76^,^77^,^78^. Recent experimental studies have shown that the LFP power in gamma band and the activities of narrow-spiking inhibitory interneurons measured from the mature V1 of awake mice were preferentially modulated by the behavioral changes^42^,^79^. Consistently, we also observed that there was a significant reduction of the gamma band SFC of narrow-spiking units in the mature V1 during the visual stimulation, while very little change of the gamma band SFC of broad-spiking units was found. The visually induced beta band SFC of the narrow-spiking units showed relatively larger increase than that of the broad-spiking units over the V1 development (Fig. 4), especially in the active states (Supplementary Fig. 9C,D). These results suggest that cortical inhibitory interneuron may play a more important role in the maturation of visually induced beta and gamma band oscillations during the V1 development. This is consistent with a large body of evidence that has suggested an experience-dependent gradual maturation of cortical inhibitory interneurons from the eye-opening to the CP^61^,^80^ and their regulatory contribution to the plasticity in the developing mammalian V1^34^,^81^. In addition to the involvement of cortical local inhibitory interneurons, our results also suggest that the experience-dependent increase of thalamocortical input strength^58^,^59^, estimated by the amplitude of VEP recorded around the layer 4, could also be involved in the emergence of visually induced beta and gamma band oscillations in the developing V1 during the CP (Figs 5 and 6 and Supplementary Figs 4 and 5).

Finally, it is interesting to consider these characteristic neuronal oscillations during the CP in relation to experience-dependent plasticity of developing V1. A recent study using similar awake mice has revealed that spike responses of most V1 cells to visual inputs are strongly modulated by behavioral states, with doubling of evoked firing rates as the animal’s behavioral state transited from quiescence (stand still) to running^42^. One of characteristic cortical activities in the latter running state is the elevated oscillatory activities in LFPs^42^. Our observed CP-specific emergence of characteristic neuronal oscillations might enhance the amplitude or signal-to-noise ratio of visual responses and thus provide a strong driver for the induction of experience-dependent plasticity during the CP. However, how the cortical neural oscillations contribute to or modulate the CP plasticity of developing V1 needs to be further investigated.

## Methods

All experiments were carried out in accordance with protocols approved by the Animal Research Advisory Committee of Shanghai Institutes for Biological Sciences, Chinese Academy of Sciences (Ref. NO. NA-100418) and the State Key Laboratory of Cognitive Neuroscience & Learning at Beijing Normal University (IACUC-BNU-NKLCNL-2013-10).

### Surgical procedure

Experiments were performed on wild-type C57BL/6 mice (both male and female) at postnatal days (P) 17–19, 26–30 and 60–90. We designed our spherical treadmill setup based on Dombeck *et al.* (2007)^41^ and Niell *et al.* (2010)^42^. Seven air inlets were attached to the bowl of an 8.125′′ (206 mm) steel ladle (McMaster-Carr). The freely rotating surface, on which the animal stood with fixed head, was provided by an 8′′ diameter Styrofoam ball (Plasteel Corporation) placed inside the bowl. The behavior of the animal was captured by a camera above the setup in part of the experiments.

Prior to recording, the animal was placed in a stereotaxic apparatus and anesthetized with isoflurane in oxygen (1–3%). Throughout surgery the body temperature was kept at 37 °C by a homeostatically controlled heating pad (RWD Life Science). Following a scalp incision, the fascia on the skull was cleared by Iodine. We applied a thin layer of cyanoacrylate (VetBond, WPI Inc.) on the cleared skull to provide a substrate to which the dental acrylic could adhere. The head-plate was then attached with dental acrylic and covered the skull excluding the opening of the head-plate. The animal was then left to recover and allowed to habituate to the recording setup. The animal quickly learned to stand still, run and even to occasionally groom.

On the day of recording, the animal was again anesthetized as described above. A craniotomy (~1 mm in diameter) was made over the binocular zone of the visual cortex (~3 mm lateral lambda) or over the thalamus (P60–90: ~2.3 mm lateral and ~2.7 mm posterior; P16–19: ~2 mm lateral and ~2 mm posterior from the bregma suture). The brain surface was covered with 1.2% agarose in saline, and then the head-plate opening was again filled with silicone elastomer (Kwik-Sil, WPI Inc.). Lastly the animal was allowed to recover for 1–2 hours before recording. The Dura was stripped off before the insertion of electrode array.

### Visual stimulation

Visual stimuli were generated with a PC computer containing a NVIDIA GeForce GT430 graphics board and displayed on a CRT monitor (Sony CPD-G520, 40.5 × 30.5 cm, 800 × 600 resolution, refresh rate 120 Hz, mean luminance of 30 cd/m^2^) placed ~20 cm in front of the animal and centered on its midline. Luminance nonlinearities were gamma corrected. We mapped the receptive fields (RFs) using sparse 2D noise stimuli, in which a white square was flashed on a black background at each of the 8 × 8 positions in a pseudorandom sequence. The 1 Hz phase reverse static gratings with different spatial frequencies (0.01, 0.02, 0.04, 0.06, 0.1, 0.2, 0.4, 0.6 cycles per degree) and 12 orientations were used to evoke the transient VEPs for characterizing the spatial frequency tuning curves. We presented standard sinusoidal drifting gratings randomly (spatial frequency: 0.02 or 0.04 cycle/deg, temporal frequency: 3 Hz, stimulus duration: 2 s, inter-stimulus interval: 2 s, gray blank with mean luminance, 12 repetitions for each orientation) to characterize the visually induced oscillatory activities.

### Electrophysiological recording

Local field potentials (LFPs) and spikes were recorded with custom-made electrode arrays (impedance 0.3 – 0.8 MΩ) and multi sites silicon probe (Model A 1 × 16 or A 1 × 32 probe, Neuronexus Technologies) placed within the binocular area of V1 or dLGN. In case of a custom-made array, four to six Ni-Cr alloy wires (California Fine Wire Company, CFW 100188) were arranged into one or two rows with an interval of 200–300 μm. In addition to using the uncoated stainless steel wire inserted in the V1 as a common reference, another reference electrode (Ni-Cr alloy wire) placed in the agarose that covered the cortex (at a position ~2 mm above the V1 surface) was additionally used to monitor and reduce the background noise especially during the animal running periods. To maximally reduce the artifact of the line noise (50 Hz), the electrode headstage and adaptor were wrapped with tin foil and grounded. In the experiments of recording muscle electrical activity (electromyogram, EMG), two electrodes were placed in neck muscles at the right and left sides, following a previous study^82^. These extra recordings in the muscle and the agarose help to rule out the potential artifacts of other activity sources in the cortical LFPs’ oscillations (Supplementary Fig. 2). Electric signals were recorded with the Cerebus™ Data Acquisition System (Blackrock Microsystems Inc.). LFP was band-pass filtered between 1–500 Hz and sampled at 2 kHz. Spiking signals were band-pass filtered between 250–7500 Hz and sampled at 30 kHz. For the spike detection, the threshold was set at about 6 times of the noise level (root mean square: RMS). Raw spikes were sorted as putative single units by using commercial software (Offline sorter) and then classified (K-means method) as broad- and narrow-spiking units based on the waveform^42^,^55^.

### Data Analysis

All data analyses were implemented with custom-written Matlab (MathWorks) scripts.

### Pre-whiten the LFP signal

The power spectrum of LFP signals typically fall-off proportional to 1/f. To reduce the dynamic range and thus reduce the leakage of low frequencies into higher frequencies during spectral estimation, we whitened the LFP signals. In the pre-whitening process, a low-order (order = 3) autoregressive (AR) spectrum estimation was used, which can reduce the dynamic range but without fitting specific structural features of the data^42^,^44^. We used the Levinson Durbin recursion method to fit the AR model, and the coefficients (A_k_) of this process were then used to filter the original time series data (X_t_). The residuals:


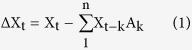


were subject to the later spectral analysis.

### Spectral analyses and brain states

We first normalized the filtered LFP signals (1–200 Hz) by calculating the z-score and performing the pre-whitening as described above. The power spectrum of the spontaneous LFP was computed using the multi-taper estimation method in Matlab with the chronux package (http://chronux.org), using 10 s data segments and 3–5 tapers (TW = 3, K = 5). Then, we smoothed the spectrum by using the “locfit” function (provided by the chronux toolbox) after removing line noise (50 Hz) in part of mice to reduce the variation but not destroy the features. For the time-frequency spectral analysis, epochs (1–10 minutes) of spontaneous recordings and 4 s visual stimulation (including the blank) were used with a 0.5 s sliding window during spectral estimation. The power was finally normalized by the mean power across all frequency bands (1–90 Hz). For characterizing the two local brain states, we calculated the LFP power ratio between high (40–70 Hz) and low (1–20 Hz) frequency bands. Then, we classified spontaneous epochs (or visual stimulation trials) into active or non-active groups if the mean power ratio during an epoch with the length of 50–300 s (or the blank of a trial) was larger or smaller than the global mean value of the whole recording (state transitions existed in near all of our recordings), respectively. This classification was verified by simultaneously recorded behavior data, in which the running and quiescent periods corresponded well to the active and non-active states of our classification criterion, respectively. The motion level (relative speed, r.s.) in the animal behavior was defined as the video frame-to-frame relative luminance change of pixels around the animal body as illustrated in Supplementary Fig. 1.

### Cross-frequency coupling (CFC)

To assess the phase-amplitude CFC we followed Canolty *et al.* (2006)^51^. LFP was first band-pass filtered using an infinite impulse response (IIR) Elliptic filter by means of the “filtfilt” function (Matlab). Then the Hilbert transform (Hilbert function, Signal Processing Toolbox) was used to compute the instantaneous beta (15–35 Hz) or gamma (40–70 Hz) amplitude, A_G_(t), and theta (3–8 Hz) phase, Φ_T_(t). A composite complex-valued signal:


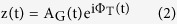


was constructed. The mean of z(t) was called M_RAW_ here. A set of surrogate composite complex-valued signals:


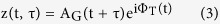


were constructed by offsetting A_G_ and Φ_T_ by some large time lag to compute {M_sur_}. The modulus of M_RAW_, compared to the distribution of surrogate modulus, provides a measure of the coupling strength, while the angle of M_RAW_ compared to the distribution of surrogate angles, suggests the preferred phase of theta with the largest gamma amplitudes. Lastly we define a normalized length of


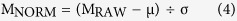


where μ is the mean of the surrogate lengths and σ is their standard deviation. This normalized M_NORM_ represents the cross-frequency coupling strength or the modulation index.

### Spike-field coherence (SFC)

The LFP filtered between 1–200 Hz and spike trains of single unit were used to calculate the spike triggered average (STA) and the SFC. For calculation of the STA, LFP segments were averaged within a window of ± 200 ms centered on each spike. After calculation of the STA, we calculated the power spectrum of each of the spike triggered LFP segments and then averaged these spectra to obtain the spike-triggered power spectrum. The SFC was then computed as the ratio of the power spectrum of the STA over the spike-triggered power spectrum^48^. The SFC in Fig. 2 was calculated with spikes during spontaneous activity. The time window of 200–1800 ms before and after the stimulus onset was used to calculate the baseline and visually induced SFC (z-score), respectively. Visually induced change of SFC was defined as:


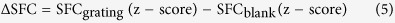


### Spike rate, VEP, spatial frequency tuning and receptive field

Mean firing rates were calculated during baseline (2 s blank) and evoked peak (within 100 ms after the stimulus onset) activity. VEP was calculated as the stimulus triggered LFPs’ average. Negative peak amplitudes of the VEPs were calculated for drifting gratings, contrast reverse static gratings and sparse 2D noise stimulation. When estimating the spatial frequency tuning curve, the VEP amplitude was normalized to that of the optimal spatial frequency (0.02 cpd). Corner frequency was defined by locating (by interpolating the raw data linearly) the frequency where the VEP amplitude dropped to 0.707 relative to that at the optimal frequency. For estimating the receptive fields, VEP evoked by bright flash in each grid and the spike triggered stimulus average in pre-spike frames were calculated.

### Current source density (CSD)

VEP was first calculated for each site of silicon probe. The CSD analysis of the VEP profiles was calculated by a three-point formula which approximates the second spatial derivative^54^:


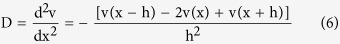


Where v is the voltage, x is the point at which D is calculated and h is the differential grid of space. The inter-contact spacing on the electrode was used as the grid (h). Linear interpolation was lastly used for smoothing.

### Statistics

Data are presented as mean ± s.e.m. unless stated otherwise. Statistical significance for all the group means of three ages were tested with the unpaired *Kolmogorov–Smirnov* test.

## Additional Information

**How to cite this article**: Chen, G. *et al.* Experience-dependent emergence of beta and gamma band oscillations in the primary visual cortex during the critical period. *Sci. Rep.* **5**, 17847; doi: 10.1038/srep17847 (2015).

## Supplementary Material

Supplementary Information
