# Supplementary material for: Experience-dependent emergence of beta and gamma band oscillations in the primary visual cortex during the critical period
Source: Sci Rep. 2015 Dec 9;5:17847. doi: 10.1038/srep17847 (PMC4673459; doi:10.1038/srep17847)
Supplement: Supplementary Information [file srep17847-s1.pdf]

Supplementary information

Experience-dependent emergence of beta and gamma band oscillations in the primary visual cortex during the critical period

Guang Chen[1,2,3], Malte J. Rasch[2], Ran Wang[2] & Xiao-hui Zhang[2,*]

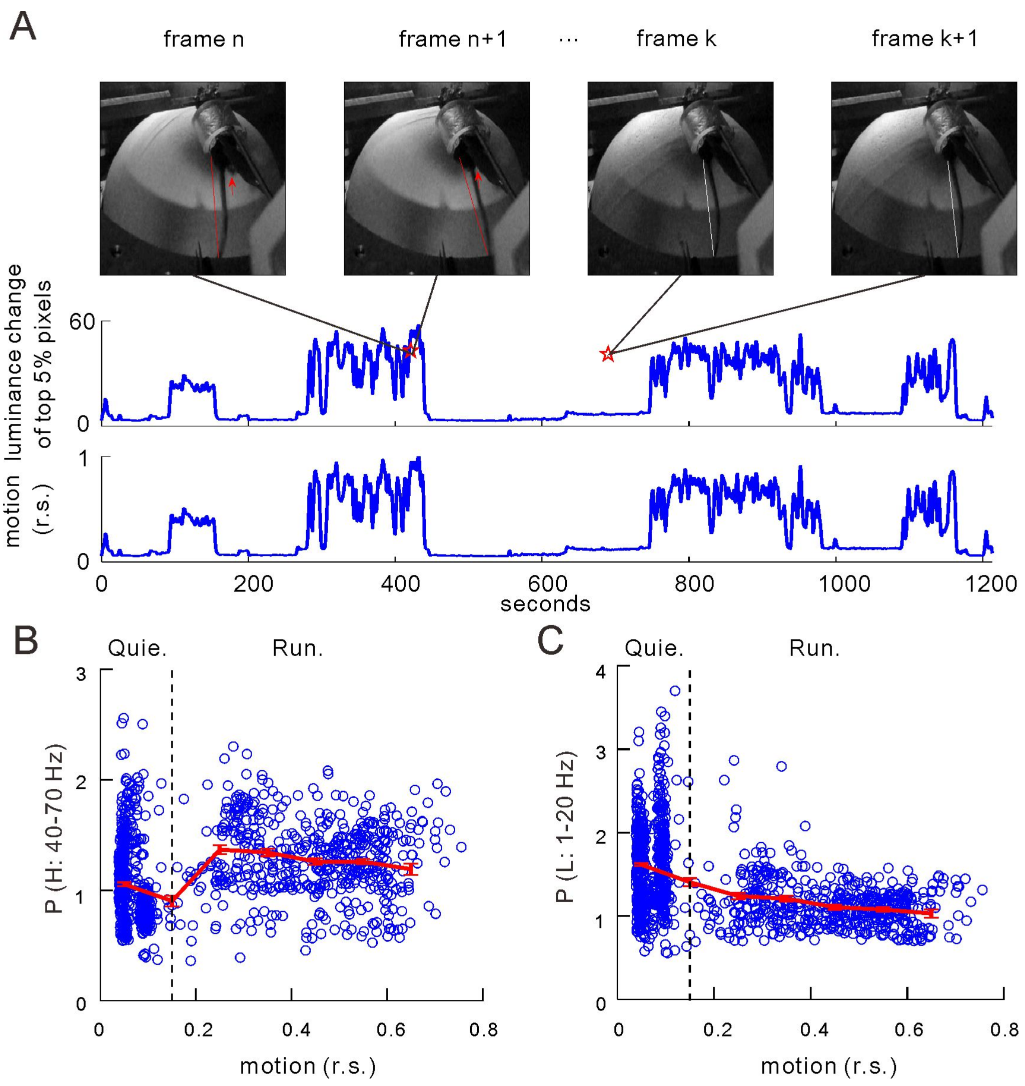

Supplementary Figure 1. Correlation between LFP power and the animal motion. (A) Procedures for calculating the mouse motion level. Video of the mouse behavior was captured (25 frames/s) by a digital camera. Luminance change of each pixel between two adjacent frames (down sample to 5 frames/s, top) was first calculated and then the mean luminance change of the top 5 % changed pixels was used as an index of the animal motion (middle). The red lines and arrows in the adjacent two frames indicate the location change of the mouse body and the corresponding luminance change was marked by the first red asterisk. The second asterisk indicate the near zero change of the second adjacent two frames. A relative speed (r.s., normalized to the maximal) was shown at the bottom. This calculation could accurately discriminate the standing still/quiescent (Quie., r.s. < 0.15) and running (Run., r.s. > 0.15) states of the animal in our experiments. (B, C) Correlation plots of the LFP power in high (H: 40-70 Hz; B) and low (L: 1-20 Hz; C) frequency bands to the mouse motion (r.s.). Solid red line represents averaged values of the power calculated with a bin width of 0.1 r.s. Error bars represent s.e.m.

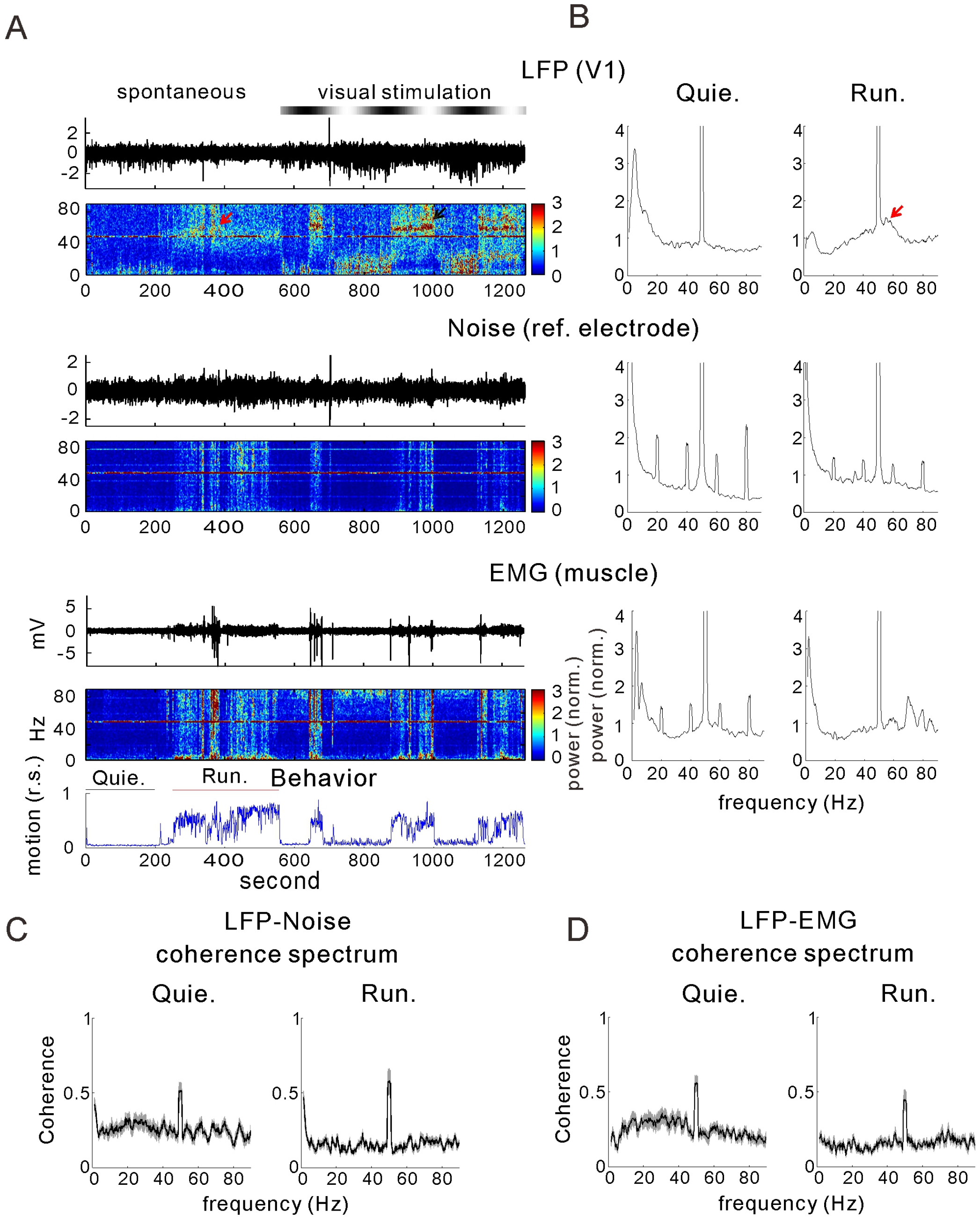

A
B
LFP (V1)
spontaneous
visual stimulation
Quie.
Run.
Noise (ref. electrode)
EMG (muscle)
mV
Hz
power (norm.)
power (norm.)
Quie.
Run.
Behavior
motion (r.s.)
second
frequency (Hz)
C
LFP-Noise
coherence spectrum
Quie.
Run.
Coherence
frequency (Hz)
D
LFP-EMG
coherence spectrum
Quie.
Run.
Coherence
frequency (Hz)

Supplementary Figure 2. Comparing the LFP, noise and muscular electrical activities. (A) The raw electrical activities (LFP, Noise and EMG) and the corresponding power spectrogram recorded from LFP electrode (placed in V1 layer 4), reference (ref.) electrode (placed in agarose with 2 mm above V1) and the electromyographical (EMG) electrode (placed in neck muscles). The animal behavior was shown at the bottom. The black and red lines indicate the periods of standing still (quiescent, Quie.) and running (Run.) states, respectively. (B) The spontaneous power spectra of LFP (top), Noise (reference electrode, middle) and EMG (bottom) activities during quiescent (Quie., left) and running (Run., right) periods. (C) The coherence spectrum between the LFP and Noise activities during quiescent (left) and running (right) periods. (D) The coherence spectrum between the LFP and EMG activities during quiescent (left) and running (right) periods. The shadowed area is trial-to-trial s.e.m. Each trial is a 3s segment of electrical activities. Coherence was calculated by the "coherencyc" function in the chronux package http://chronux.org.

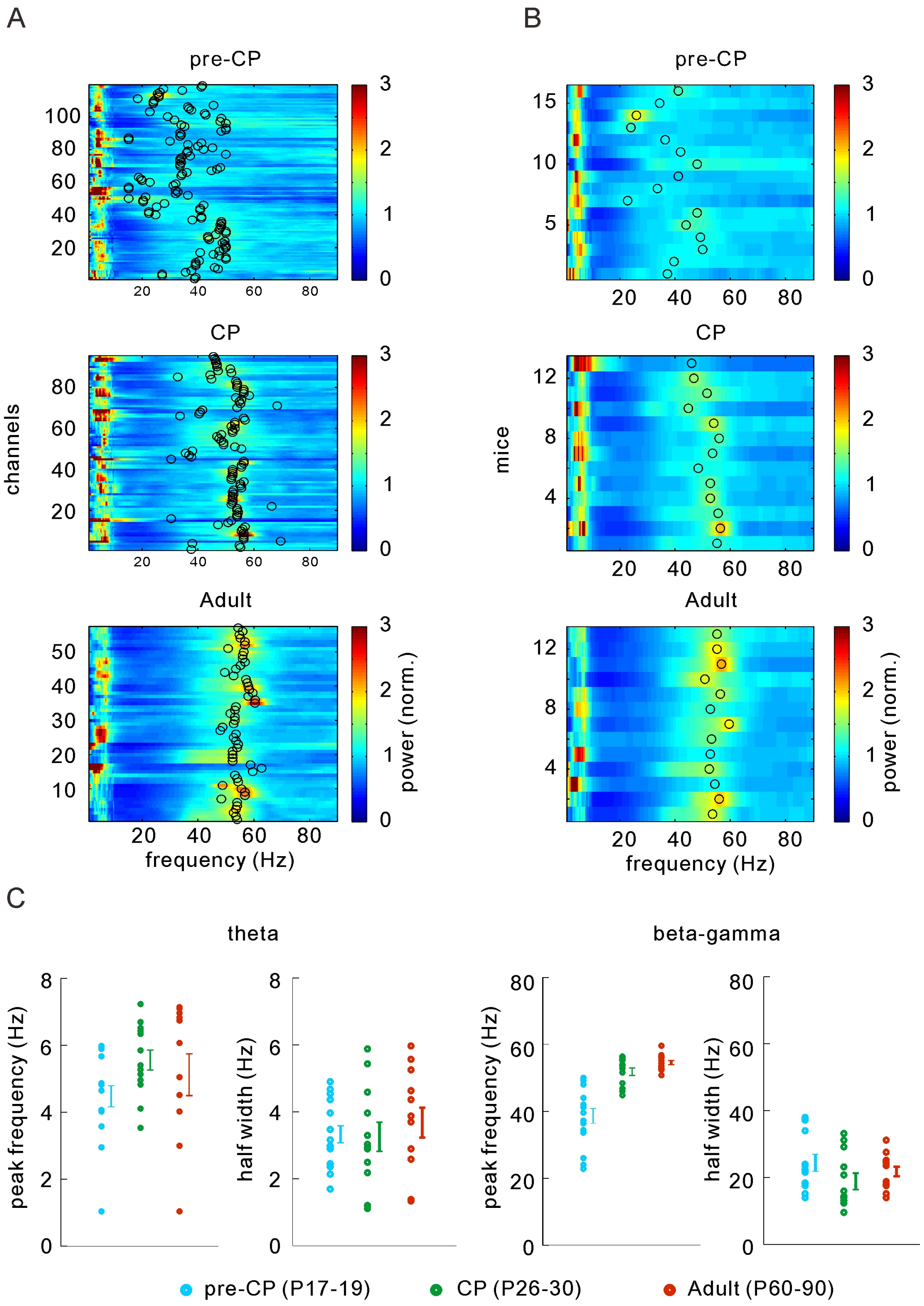
A
B
pre-CP
CP
Adult
channels
mice
frequency (Hz)
power (norm.)
C
theta
beta-gamma
peak frequency (Hz)
half width (Hz)
pre-CP (P17-19)
CP (P26-30)
Adult (P60-90)

Supplementary Figure 3. Characteristics of the spontaneous LFP power spectra. (A) The spontaneous LFP power spectra during active state of all recording channels from mice at three different ages. (B) The averaged spontaneous LFP power spectra across channels in each mouse during active state at three different ages. The black circles indicate the peak frequencies of the beta-gamma band power spectra. (C) The distribution of theta and beta-gamma band peak frequencies and half peak widths of the power spectra of all mice. Error bars represent mouse-to-mouse s.e.m.

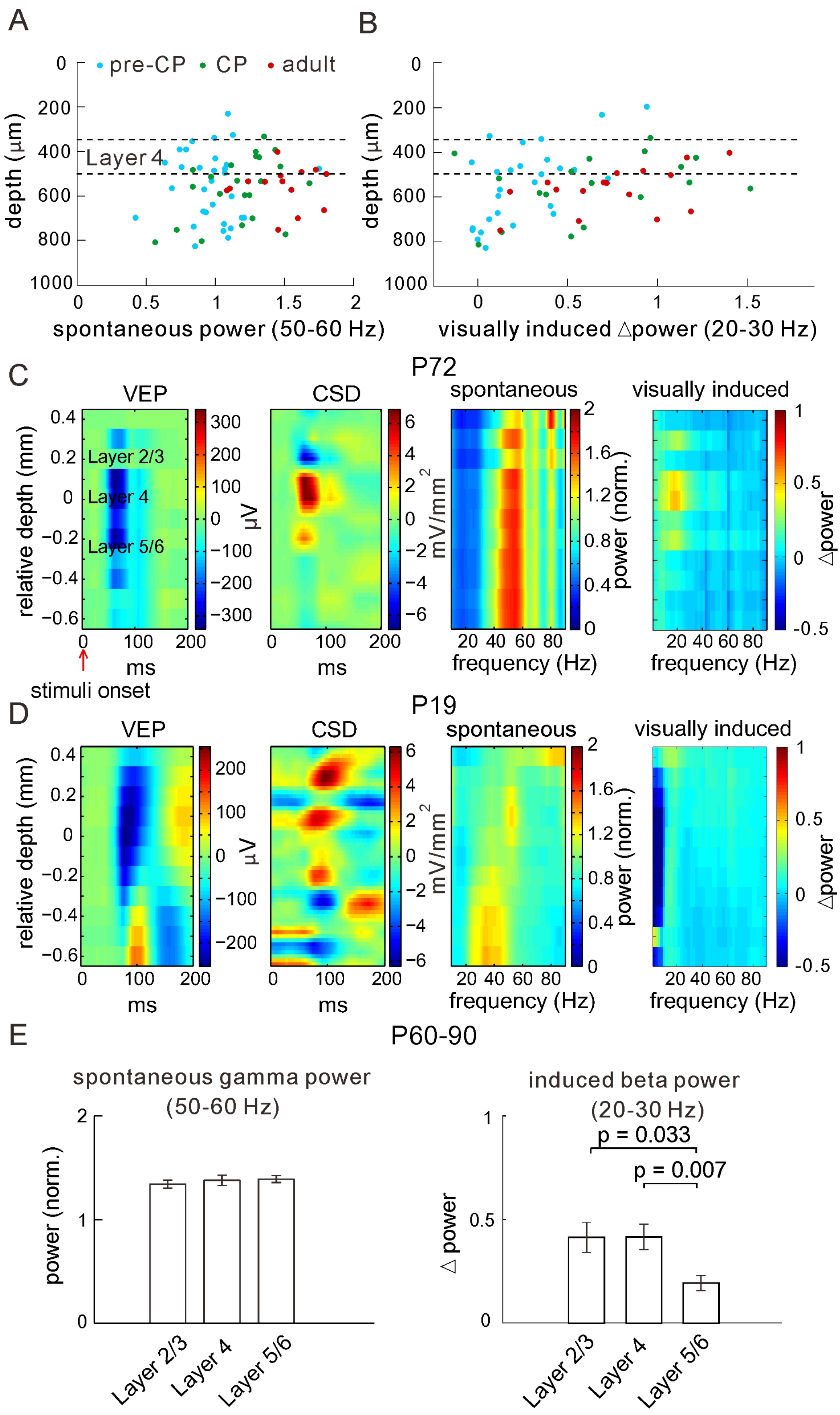
A
pre-CP
CP
adult
depth (µm)
Layer 4
spontaneous power (50-60 Hz)
B
depth (µm)
visually induced Δpower (20-30 Hz)
C
P72
VEP
CSD
spontaneous
visually induced
relative depth (mm)
Layer 2/3
Layer 4
Layer 5/6
µV
mV/mm2
power (norm.)
Δpower
ms
stimuli onset
frequency (Hz)
D
P19
VEP
CSD
spontaneous
visually induced
relative depth (mm)
µV
mV/mm2
power (norm.)
Δpower
ms
frequency (Hz)
E
P60-90
spontaneous gamma power
(50-60 Hz)
power (norm.)
Layer 2/3
Layer 4
Layer 5/6
induced beta power
(20-30 Hz)
p = 0.033
p = 0.007
Δ power
Layer 2/3
Layer 4
Layer 5/6

Supplementary Figure 4. Cortical layer distribution of spontaneous and visually induced LFP activities. (A, B) The distribution of recording depths in relation to the spontaneous gamma band power (A) or the visually induced changes of beta band power (B). (C) Example silicon probe recording in an adult mouse. VEPs of recording sites at different depths and the corresponding current source density (CSD) were shown (left). LFPs' spontaneous power spectra and visually induced power changes at different depths were calculated (right). (D) Same as C but for the recording in a pre-CP mouse. (E) Comparison of the spontaneous gamma power and visually induced beta power between different layers of all silicon probe recordings in adult mice ($n = 7$ probe recordings in 7 mice). Error bars represent s.e.m. The p values were calculated by the unpaired *Kolmogorov-Smirnov* test.

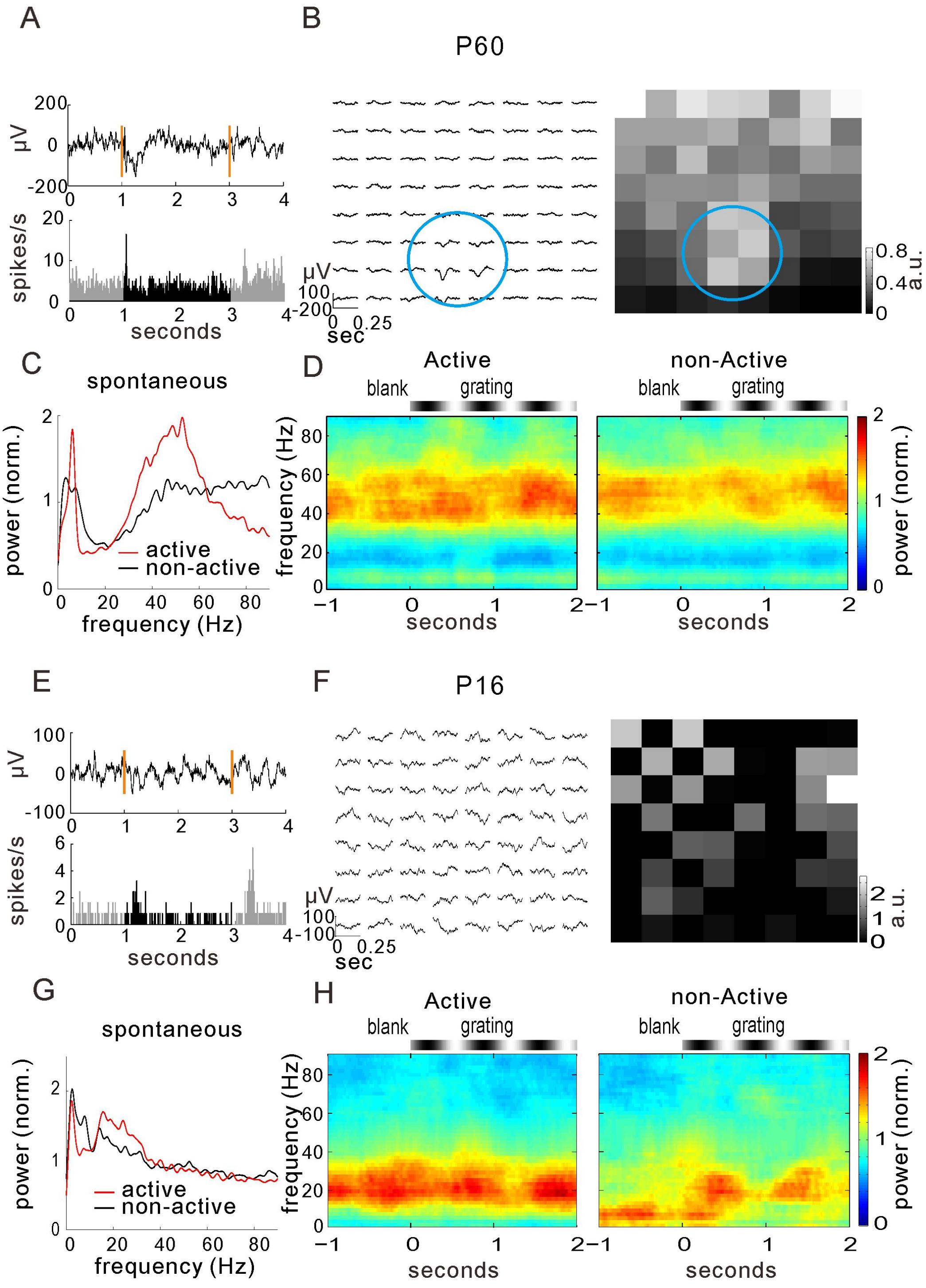

A
B
P60
μV
spikes/s
seconds
μV
100
-200
0 0.25
sec
0.8
0.4
0
a.u.
C
spontaneous
power (norm.)
active
non-active
frequency (Hz)
D
Active
non-Active
blank
grating
frequency (Hz)
seconds
power (norm.)
E
F
P16
μV
spikes/s
seconds
μV
100
-100
0 0.25
sec
2
1
0
a.u.
G
spontaneous
power (norm.)
active
non-active
frequency (Hz)
H
Active
non-Active
blank
grating
frequency (Hz)
seconds
power (norm.)

Supplementary Figure 5. LFP activities in dLGN. (A) LFP and spike responses to drifting grating stimulation of an example recording site and one spiking unit in the dLGN of an adult mouse. Yellow lines indicate the onset (left) and offset (right) of the visual stimulation. (B) Receptive field measured by VEP (left) and spikes (spike-triggered stimulus average, right) with 8×8 sparse noise stimulation. Blue circles indicate the receptive field location of the same recording in A. (C) Spontaneous LFP power spectrum of the example recording during active and non-active states. (D) The visually induced power spectrogram of the same recording during active and non-active states. Similar activities have been observed in 8 mice. (E-H) Same as A-D but for an example recording in a mouse at pre-CP. Beta but not gamma band power dominates the spontaneous fast oscillatory activities in LFP, this is similar with that in V1 of mice at pre-CP. Similar activities have been observed in 2 mice.

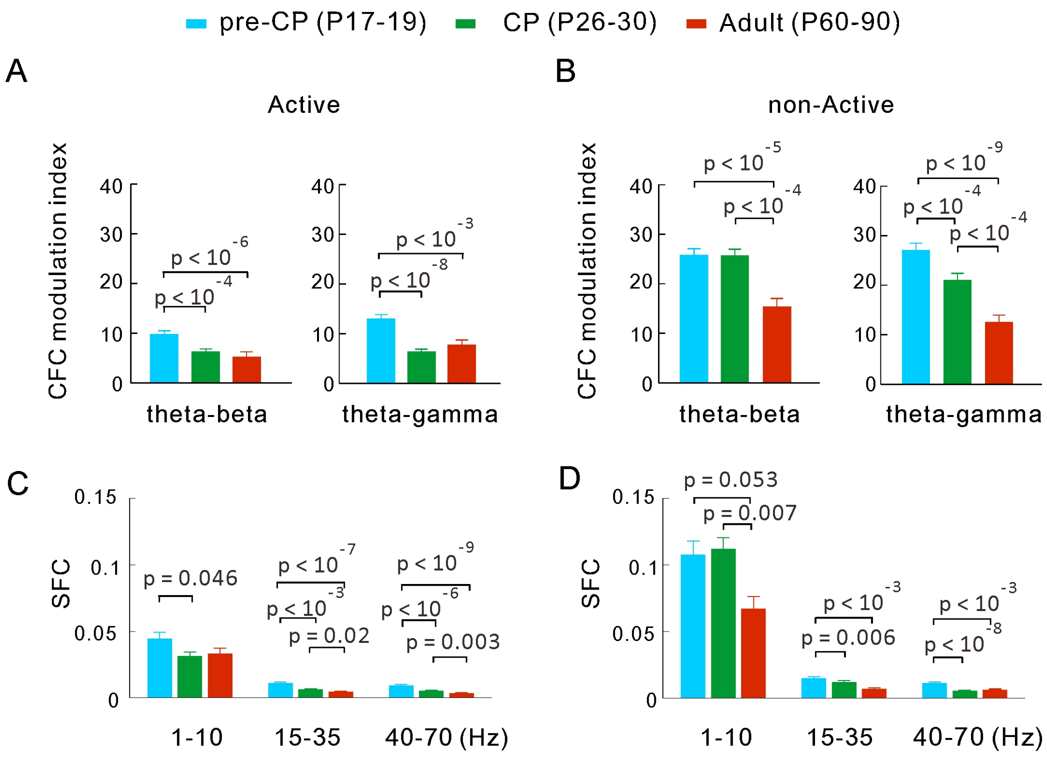

Supplementary Figure 6. CFC and SFC of spontaneous activity during Active and non-Active states. (A, B) Summarized results of the developmental changes of theta-beta/gamma phase-amplitude CFC modulation index during spontaneous activity for active (A) and non-active states (B). (C, D) Quantification of the developmental changes of SFC in three frequency bands during spontaneous activity for active (C) and non-active states (D). Error bars represent s.e.m. The p values were calculated by the unpaired *Kolmogorov-Smirnov* test.

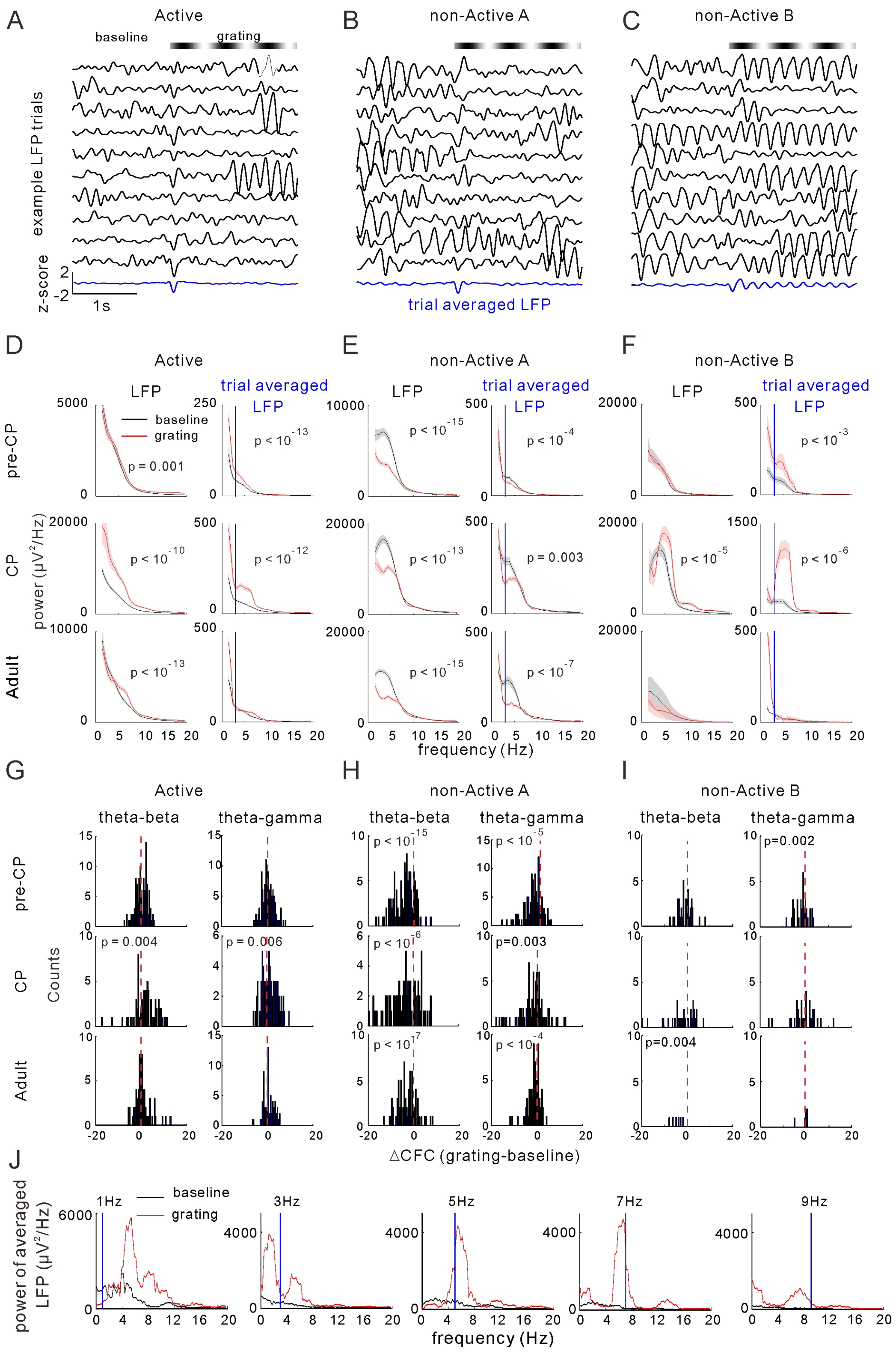

A
Active
baseline
grating
example LFP trials
z-score
1s
B
non-Active A
trial averaged LFP
C
non-Active B
D
E
F
LFP
trial averaged LFP
baseline
grating
pre-CP
CP
Adult
power (μV²/Hz)
frequency (Hz)
G
H
I
theta-beta
theta-gamma
Counts
ΔCFC (grating-baseline)
J
power of averaged LFP (μV²/Hz)
1Hz
3Hz
5Hz
7Hz
9Hz
frequency (Hz)

Supplementary Figure 7. Visual modulation to the LFP low frequency band activities and the CFC. (A-C) Example LFP segments (black) corresponding to visual stimulation trials and the averaged LFP (blue) over trials before (baseline) and during the presentation of the drifting gratings during active (A) and non-active (B, C) states. (D-F) Comparison between the baseline and visually induced low frequency (1-10) band power of raw (left) and trial-averaged LFPs (right) during active and two non-active states at three ages. The non-active A type has no significant increase of low frequency band oscillation in the trial averaged LFP (B, bottom), whereas the non-active B type has this increased oscillation in the trial-averaged LFP (C, bottom) because of the stimulus onset locking of the endogenous theta oscillation. Fractions of non-active B type recording channels were: pre-CP, 19%, 30 out of 158 in 19 mice; CP, 18%, 32 out of 180 in 16 mice; Adult, 4%, 6 out of 134 in 19 mice. The location of blue lines indicate the temporal frequency (3 Hz) of the drifting gratings. Solid lines represent the population average power spectra and shadow areas represent the recording channel-to-channel s.e.m. p values indicate significant ($p < 0.05$) statistical differences between baseline and induced power in the theta band (3-8 Hz). (G, H, I) Histogram distributions of the visually induced theta-beta and theta-gamma phase amplitude CFC changes (grating-baseline) of all recording channels during active (G) and two non-active states (H, I) at three ages. Red dash lines indicate the location of zero. (J) Comparing the modulation to LFP low frequency band power (only LFP trials with power increase were included) by drifting gratings with different temporal frequencies (data averaged from 4 recording channels in 2 adult mice). Locations of blue lines indicate the stimuli's temporal frequencies. All the p values were calculated by the paired (grating *vs* basline) *student t* test.

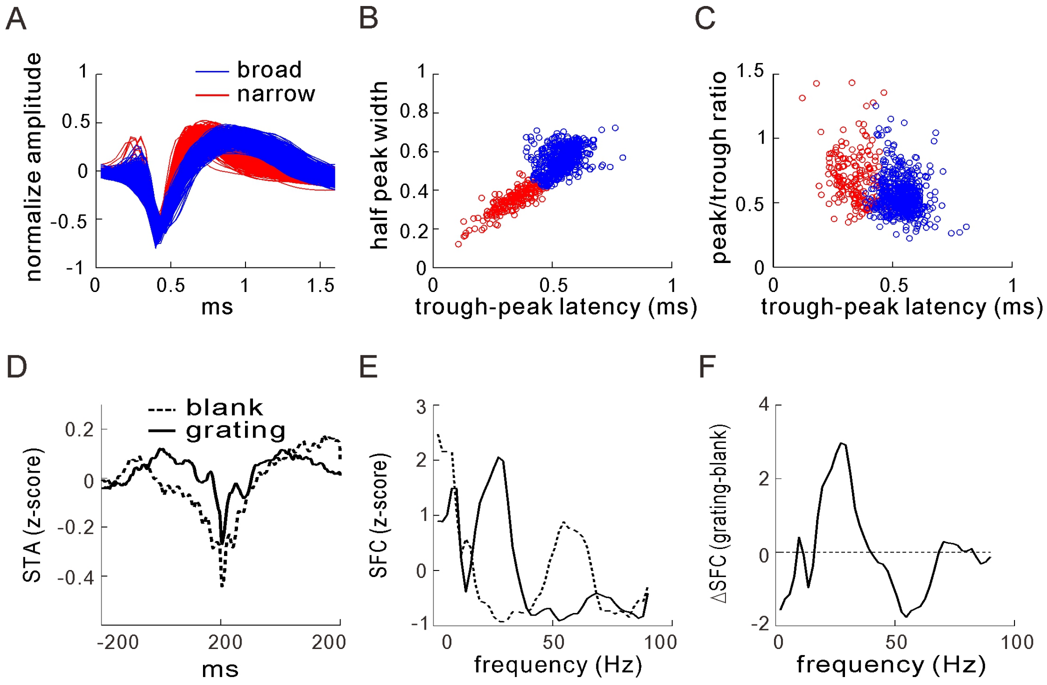

Supplementary Figure 8. Characterization of broad- and narrow-spiking units and the calculation of SFC. (A) Spike waveforms of broad- and narrow-spiking units. (B, C) K-means classification based on the features, trough-peak latency to half peak width (B) or peak/trough ratio (C), of spike waveforms. (D, E) Calculation of the spike-triggered LFP average (STA, z-score) and the corresponding spike-field coherence (SFC, z-score) of an example single unit before (blank) and during (grating) the visual stimulation. (F) The visually induced change of the SFC (ΔSFC: grating-blank).

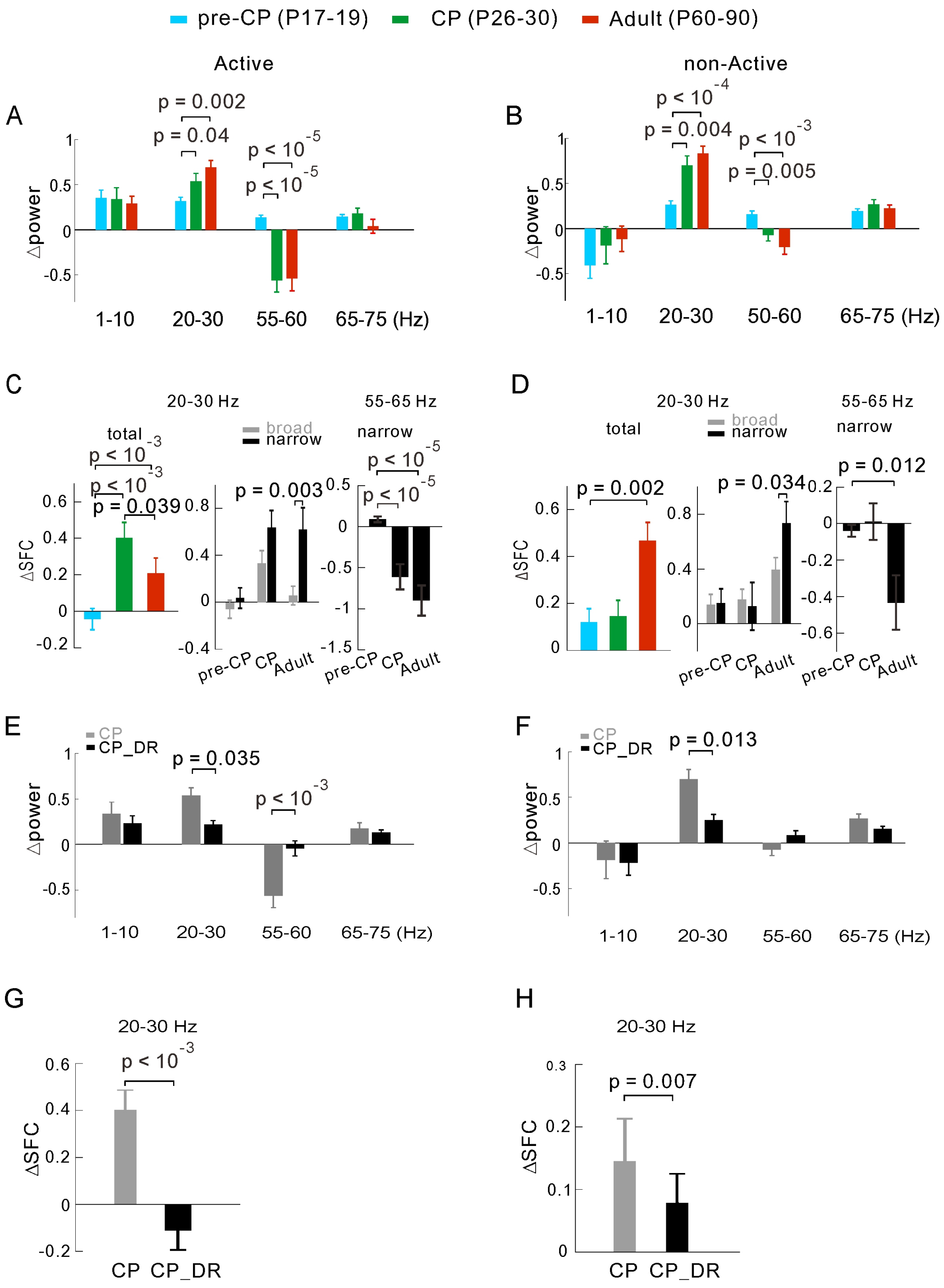
pre-CP (P17-19)
CP (P26-30)
Adult (P60-90)
Active
non-Active
A
B
C
D
E
F
G
H
Δpower
ΔSFC
1-10
20-30
55-60
50-60
65-75 (Hz)
p = 0.002
p = 0.04
p = 0.004
p = 0.005
20-30 Hz
55-65 Hz
total
broad
narrow
p = 0.039
p = 0.003
p = 0.034
p = 0.012
pre-CP
CP
Adult
CP_DR
p = 0.035
p = 0.013
p = 0.007

Supplementary Figure 9. Visually induced activities during active and non-active states. (A, B) Summarized results of the developmental changes of visually induced power change for active (A) and non-active states (non-active B type data in Supplementary Fig. 7 were not included) (B). (C, D) Summarized results of the developmental changes of the visually induced SFC change for active (C) and non-active states (D). (E, F) Comparison of the visually induced power change between dark-reared and normal-reared mice for active (E) and non-active states (F). (G, H) Comparison of the visually induced SFC change between dark-reared and normal-reared mice for active (G) and non-active states (H). Error bars represent s.e.m. The p values were calculated by the unpaired *Kolmogorov-Smirnov* test.

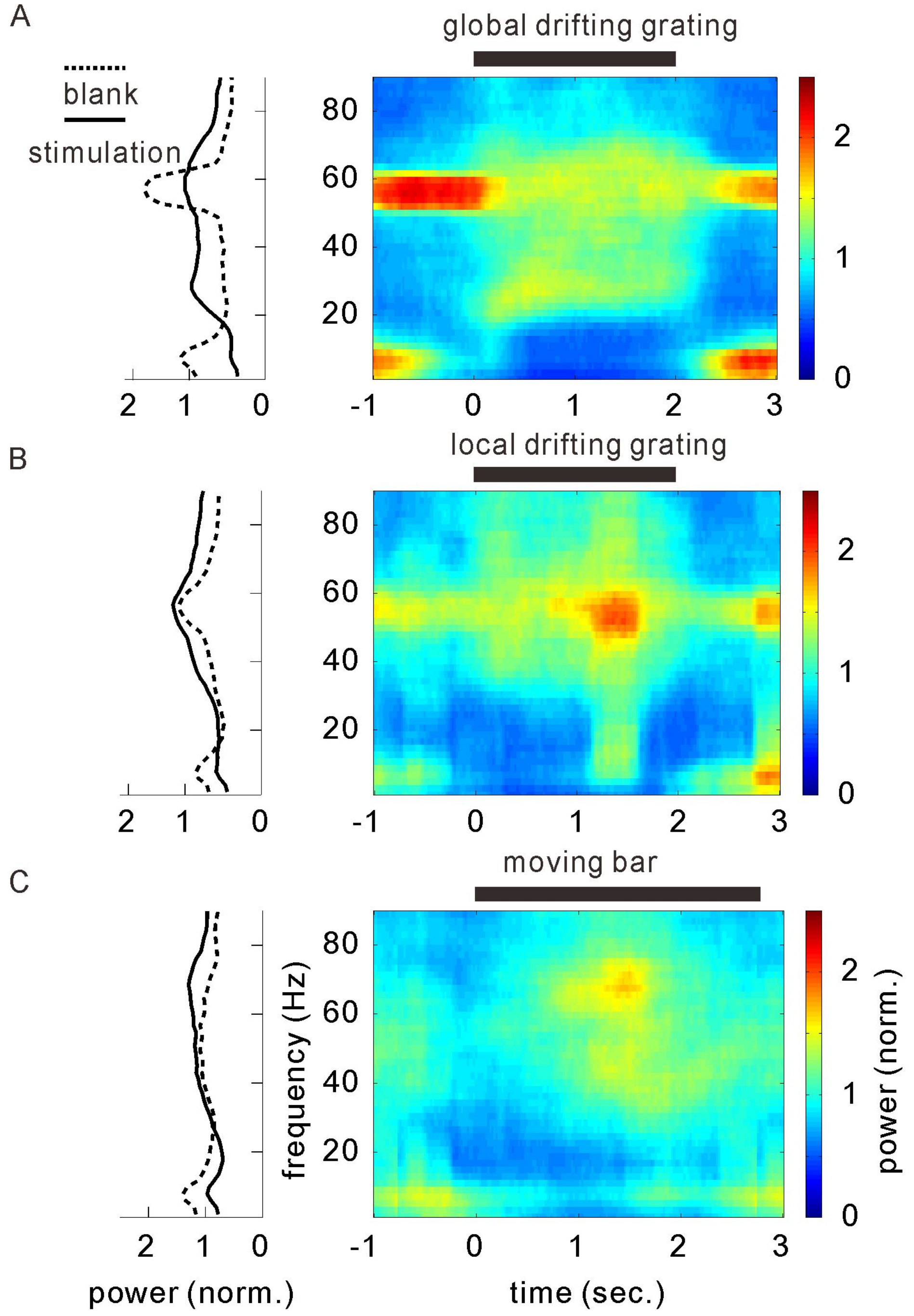
A
global drifting grating
blank
stimulation
80
60
40
20
2
1
0
2
1
0
-1
0
1
2
3
B
local drifting grating
80
60
40
20
2
1
0
2
1
0
-1
0
1
2
3
C
moving bar
frequency (Hz)
80
60
40
20
power (norm.)
2
1
0
2
1
0
-1
0
1
2
3
power (norm.)
time (sec.)

Supplementary Figure 10. Comparing the visually induced changes of the LFP power spectrum by distinct stimulus types. (A-C) Visually induced changes of the LFP power spectrum by the full-field drifting gratings (A, covering the animal's visual field with 90 degrees), local drifting gratings (B, covering the receptive field of the recording site with about 30 degrees) and moving bars (C, width of 4 degrees and length of full-screen size).
